# A serious game for speech training in dysarthric speakers with Parkinson's disease: Exploring therapeutic efficacy and patient satisfaction

**DOI:** 10.1111/1460-6984.12722

**Published:** 2022-03-26

**Authors:** Mario Ganzeboom, Marjoke Bakker, Lilian Beijer, Helmer Strik, Toni Rietveld

**Affiliations:** ^1^ Centre for Language Studies Radboud University Nijmegen Nijmegen the Netherlands; ^2^ Centre for Language and Speech Technology Radboud University Nijmegen Nijmegen the Netherlands; ^3^ iXperium/Centre of Expertise Learning and Teaching with ICT HAN University of Applied Sciences Nijmegen the Netherlands; ^4^ Research Department Sint Maartenskliniek Nijmegen the Netherlands; ^5^ Chair Key Factors in Physiotherapy and Allied Healthcare HAN University of Applied Sciences Nijmegen the Netherlands; ^6^ Donders Institute for Brain, Cognition and Behaviour Radboud University Nijmegen Nijmegen the Netherlands

**Keywords:** dysarthria, efficacy study, eHealth, serious gaming, speech training, speech intelligibility

## Abstract

**Background:**

The increasing need for speech therapy due to our ageing population raises the demand on therapeutical resources. To meet this demand, innovative delivery of speech training is required. eHealth applications may provide a solution, as intensified and prolonged training is only possible and affordable in patients’ home environment.

**Aims:**

This study explores the effects on speech intelligibility of game‐based speech training that provides automatic feedback on loudness, pitch and pronunciation. Additionally, we investigate how satisfied patients are with the game‐based speech training and how they experience the automatic feedback. Furthermore, patients’ preferences for game‐based speech training compared with face‐to‐face training are explored.

**Methods and Procedures:**

Eight adult dysarthric speakers with Parkinson's disease (PD) completed a 4‐week game‐based speech training in their home environment. For each speaker, 24 speech utterances were audio recorded 4 weeks before (T1), immediately before (T2) and immediately after (T3) the training. All speech samples were rated on speech intelligibility by 10 untrained listeners, by comparing them with the corresponding utterances realized by a healthy speaker. Changes over time were analysed using a linear mixed‐effects analysis. Patient satisfaction with the game and the automatic feedback was assessed using a questionnaire. The preferences of patients were collected using a paired comparisons procedure in which the patients were asked whether they would prefer game‐based or face‐to‐face speech training in four hypothetical scenarios with different hypothesized levels of speech improvement.

**Outcomes and Results:**

While there was no significant difference in speech intelligibility ratings between T1 and T2, we did find one between T2 and T3. At T3, speech intelligibility was rated higher than at T2, indicating positive effects of the game‐based speech training. Patients generally seemed satisfied with the game as average ratings were above 7 on a 10‐point rating scale. Generally, patients agreed with the automatic feedback and could use it to positively change the way they spoke. Patients prefer the training that provides the highest hypothetical improvement, and thus do not prefer face‐to‐face above game‐based therapy.

**Conclusions and Implications:**

The results of this study suggest that dysarthric speakers due to PD see game‐based speech therapy as a valid alternative for face‐to‐face therapy and that it leads to an average improvement in speech intelligibility. For an optimal effect and user satisfaction it should preferably not be used in isolation but in combination with face‐to‐face training. In this manner, the strengths of both therapeutic deliveries can be harnessed.

**WHAT THIS PAPER ADDS:**

**What is already known on this subject**

Dysarthric speech in patients with PD is known to benefit from intensified and long‐term speech therapy. The increasing need for speech therapy due to our ageing population raises the demand on therapeutical resources making highly frequent and long‐term therapy difficult. eHealth provides the opportunity to intensify and prolong speech training in patients’ home environment. A drill‐and‐practice method was employed and investigated in a web‐based speech application, indicating positive effects on speech intelligibility. However, participants indicated a lack of variation in exercises making the training less enjoyable. Other research showed that serious games can increase enjoyment during training.

**What this paper adds to existing knowledge**

The results of this study show that it is possible to develop a serious game that can be successfully used for speech training by patients with dysarthria due to PD. Comparing speech intelligibility ratings before and after training, we observed significant improvements in speech intelligibility ratings. Patients generally agree with the automatic feedback and can use it to positively change the way they speak. Average ratings were above 7 on a 10‐point scale, indicating that patients are satisfied with the game. Patients prefer the type of training (game based or face to face) that provides the highest hypothetical improvement.

**What are the potential or actual clinical implications of this work?**

The results of this study suggest that game‐based speech training can improve speech intelligibility in patients. This indicates that it can be considered a suitable approach in the treatment of patients with dysarthria due to PD. Patients do not prefer game‐based training above face‐to‐face training in all scenarios. For that reason, for every patient one should carefully consider how to optimally combine game‐based and face‐to‐face training.

## INTRODUCTION

The application of eHealth for patients with acquired neurological diseases has been gaining interest in the field of rehabilitation for the last two decades. eHealth is ‘the cost‐effective and secure use of information and communications technologies in support of health and health‐related fields, including health‐care services, health surveillance, health literature, and health education, knowledge and research’, (World Health Organization (WHO), 2005, p. 121). It provides potential for the increased recovery of motor skills required for mobility and speech (Beijer et al., 2014), which are frequently affected due to neurological conditions such as Parkinson's disease (PD). In close to 90% of patients with PD, motor skills involved with speech are affected, resulting in dysarthria (Moya‐Galé & Levey, 2019). Dysarthric speech is often characterized by impaired articulation and decreased voice intensity (De Bodt et al., 2002), negatively impacting speech intelligibility and hindering daily communication.

Dysarthric speech in patients with PD is known to benefit from intensified and long‐term training (Ramig et al., 2001). The benefits of short‐term intensive speech training have also recently been studied (Mendoza Ramos et al., 2021) with some encouraging results. The current standard practice for dysarthria therapy in the Netherlands is the Pitch Limiting Voice Treatment (PLVT; Kalf et al., 2011), an intensified long‐term training program. In line with the Lee Silverman Voice Treatment (LSVT; Ramig et al., 2001), the PLVT focuses on increasing voice intensity. The resulting increase of articulatory effort and precision beneficially affects speech intelligibility (Sapir et al., 2007; Wight & Miller, 2015). However, a potential side effect is that patients often raise their pitch and laryngeal muscle tension as well. Therefore, the PLVT, unlike the LSVT, focuses on increasing voice intensity while keeping the pitch at a low and comfortable level. PLVT's goal ‘speak loud and low’ is known to positively affect voice intensity in patients with PD (De Swart et al., 2003).

As described by the Dutch government, the burden put on healthcare increases every year (Van Vilsteren et al., 2022). From this, a disbalance between speech therapeutical resources and the need for speech therapy may emerge that does not allow for highly frequent and long‐term therapy, leading to a potential deprivation of optimal speech rehabilitation. As a consequence, dysarthric patients experience detrimental effects of low speech intelligibility and are highly motivated for practising their speech to support them in regaining societal participation.

eHealth provides the opportunity to both intensify and prolong speech motor training in patients’ home environment. This allows self‐management of speech training and, hence, the long‐term improvement and maintenance of enhanced speech intelligibility. Nevertheless, the absence of a therapist, providing instant feedback and a therapeutic relationship, and the need for patients to operate an eHealth device, may result in barriers for effective speech training. It is therefore vital to investigate the efficacy of eHealth‐based speech training before investigating how such barriers can be removed. The importance of providing evidence for eHealth‐based speech training in patients with acquired neurological diseases is even more obvious given the current Covid‐19 pandemic (2020–22), which demands physical (‘social’) distance between therapists and their patients for an—as yet—unknown period of time.

There are various ways of practising speech through eHealth. A drill‐and‐practice method was employed and investigated in a web‐based speech application (Beijer et al., 2014). Data in that study indicated beneficial effects on speech intelligibility and user experience. However, a key point of feedback provided by the patients was the lack of variation in speech training exercises and the poor ecological validity of the application. That is, they reported being able to follow the program, but did not experience beneficial effects in daily‐life communication. A plausible explanation is that the drill‐and‐practice nature of the exercises provided by the application may lack the transfer of the improved speech skills to daily‐life situations. Also, a drill‐and‐practice approach challenges patients’ therapy compliance, which is a key requirement for the maintenance of regained speech intelligibility. In this respect, it is worthwhile to explore alternative approaches for remote and independent speech training. One such approach is the use of serious games. A widely accepted definition of serious games is: ‘games that do not have entertainment, enjoyment, or fun as their primary purpose’ (Laamarti et al., 2014, p. 3). In many cases, entertainment, enjoyment or fun is used to serve the primary purpose of a serious game. For example, previous research showed that serious games can increase enjoyment during training (Lewis et al., 2016; Kari, 2017), and have the potential to trigger patients’ intrinsic motivation for therapeutic practice, enhancing therapy compliance as a result. Additionally, compared with more drill‐and‐practice eHealth applications, serious games allow speech training in ecologically valid scenarios that are more suitable to transfer the improved speech skills to daily‐life situations. For example, a scenario in which a patient needs to converse with another person. Given these potentials, the current study continues our exploration of how serious games can be used to improve patients’ speech intelligibility.

In our project ‘Challenging Speech Training in Neurological Patients by Interactive Gaming’ (CHASING) a serious game (Treasure Hunters) for patients with dysarthria due to PD or stroke was developed and evaluated. The goal of this game was to improve speech intelligibility in functional communication of these patients (Ganzeboom et al. 2016). In our previous study (Ganzeboom et al., 2018), we explored the added value of game‐based speech training (using the game Treasure Hunters) compared with non‐game computer‐based speech training. Treasure Hunters was a two‐player cooperative game in which players navigated a virtual map and needed to help each other to find the treasure by exchanging information via speech. The game provided automatic feedback on loudness and pitch in accordance with PLVT prescriptions. The results of our previous study (Ganzeboom et al., 2018) were mixed. No clear evidence of the efficacy of the game‐based speech training was found and there was substantial variation in user satisfaction. In our continued aim to find a modality in serious gaming with clear evidence of efficacy for the patients in question, an adapted version of Treasure Hunters was developed, called Treasure Hunters 2, which was evaluated in a different experimental design. The effects of the improved game‐based speech training were compared with a period of no training instead of a different type of speech training. For the current study, we hoped to encourage more participants to join as they only had to follow one speech training program. Treasure Hunters 2 was redesigned to increase the intensity of the speech training by including game elements that required the pronunciation of longer utterances. Pronunciation exercises were also introduced in the game to address the difficulties dysarthric speakers have with articulation. In addition to automatic feedback on voice intensity and pitch, feedback on players’ pronunciation was automatically provided by the automatic speech recognition (ASR) technology developed for this study.

We assessed the effects of our improved speech training on different aspects. First, how the game affected speech intelligibility. Second, patient satisfaction with the improved game design was a critical component. In addition, we sought to gauge patient experience and interpretation of the new feedback visualization for loudness, pitch and pronunciation. A final critical area of focus was patient preference for game‐based speech training as compared with traditional face‐to‐face training. These interests led us to address the following four research questions:

- Can a game‐based speech training positively affect speech intelligibility in patients with dysarthria due to PD?
- How satisfied are patients with dysarthria due to PD with game‐based speech training?
- How do patients with dysarthria due to PD experience the feedback on pitch, loudness and pronunciation in a game‐based speech training?
- Do patients with dysarthria due to PD prefer game‐based speech training to traditional face‐to‐face training?

## TREASURE HUNTERS 2

Treasure Hunters 2 is based on PLVT and aims to provide intensive speech training focusing on increasing loudness at a low pitch, and improving articulation. It has been developed in collaboration with Creative Care Lab at Waag Society (http://waag.org/project/chasing, http://hstrik.ruhosting.nl/CHASING/). The game targets elderly patients, because this group is largely representative of the population of patients with dysarthria due to PD. Both patients and speech therapists were involved at multiple stages of the game design and development process.

### Game design

To integrate the practices of the PLVT, a serious game was designed that encouraged players to speak continuously and engage in highly frequent training. Additionally, it provided feedback that motivated players to speak louder at a low pitch and improve their articulation. In our previous research (Ganzeboom et al., 2016), we described that a cooperative game design facilitates intensive speech training. Players need to help each other to reach a common goal and, consequently, are encouraged to speak to each other continuously. Like the previous version of our game, Treasure Hunters 2 is also a two‐player cooperative game. Players participated in different stories, such as one in which they are archaeologists searching for specific objects or another in which they are detectives tasked with solving a crime. Players saw themselves walking on a map and often did not see the other player immediately. They needed to describe their location and their surroundings to be able to help each other, encouraging them to speak. To increase the intensity of the training further, the game elicited longer utterances by requiring players to find their own specific clues that they needed to share verbally. Some clues hinted at how one player could help the other find the next clue, thus facilitating the need for sharing. Additionally, the element of opening gates or doors by correctly reading a sentence aloud was added as an exercise to train players’ articulation. That was also beneficial to increasing the amount of longer utterances elicited by the game. Players played the game at a distance over the internet and spoke to each other via a voice chat connection.

### Feedback on voice loudness and pitch

**FIGURE 1:**
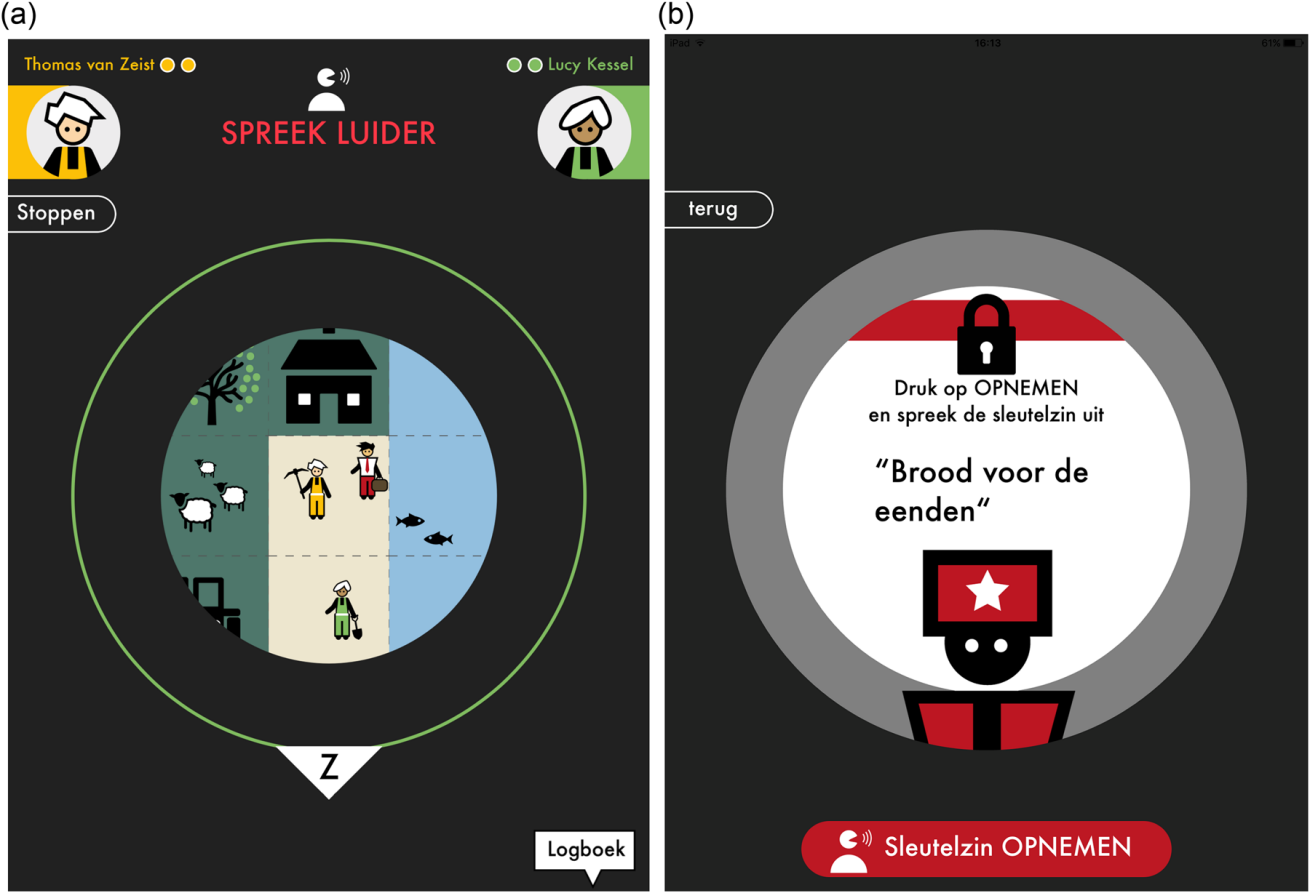
Screenshots of the game: (a) ‘speak louder’ (Dutch: *Spreek luider*) notification in red including the green circle showing to what extent the view on the playing field can grow when speaking louder; and (b) the initial screen of the pronunciation exercise with, at the centre, the sentence to speak aloud: ‘Bread for the ducks’ (*Brood voor de eenden*) and the red button at the bottom to start and stop recording the exercise [Colour figure can be viewed at wileyonlinelibrary.com]

While playing, the players continuously received automatic feedback on voice loudness and pitch by means of speech analysis algorithms. We merged the feedback on voice loudness with the circular shaped view of the playing field to have it in the focus of players’ attention. The view grew larger and smaller depending on whether the player spoke loud enough or too soft. This way, we aimed to intrinsically motivate players to speak loud enough by rewarding them with a larger view of the playing field making more of the immediate environment around their character visible. Figure 1a shows the view on the playing field and the green circular shaped line to which it can grow. In order to add a more direct way of feedback, a ‘speak louder’ notification was added above the view. The ‘speak louder’ (Dutch: *Spreek luider*) notification is shown in red in Figure 1a. Feedback on pitch is provided exclusively via a ‘speak lower’ (*Spreek lager*) notification at the same location. It is shown in blue to ease its identification.

Thresholds were used to determine when feedback on loudness and pitch should be given: for male and female participants, initial levels of intensity were set at 60 decibel or above (Rietveld and Van Heuven 2016). When participants’ intensity stayed above this threshold their view of the playing field grew. Oppositely, it started shrinking to its minimum size when the intensity moved below the threshold. When this occurred, the ‘speak louder’ notification also showed. Initial levels for pitch were set below 170 Hz for male participants and below 260 Hz for female participants (Kalf et al., 2011). When participants raised their pitch above the threshold, the ‘speak lower’ notification was shown. These values were additionally calibrated within the game with respect to environmental noise in a preparatory session in participants’ home environment.

### Feedback on pronunciation

In addition to feedback on loudness and pitch, Treasure Hunters 2 also provided feedback on pronunciation. Players had to read aloud a phrase to open a gate or door in the game and received feedback at word level on potential mispronunciations. We chose to provide feedback at word level assuming that feedback at a more detailed level (e.g., syllable or phoneme) may be too difficult for players to understand and interpret in a gaming environment. Players had three attempts in total to correct any detected mispronunciations. After the third attempt, the gate opened automatically in order to not hinder the continuity of play. The screen starting the pronunciation exercise is shown in Figure 1b. It shows a short instruction at the top of the circle on how the player should proceed and the sentence to speak aloud in the middle, ‘Bread for the ducks’ (*Brood voor de eenden*). After having read the sentence in silence (to prevent possible hesitations), the player started the recording by pressing the red button at the bottom, and stopped the recording by pressing the same button again. Afterwards, the feedback screen displayed words in which a mispronunciation was detected in red, and words in which none was detected in green. Specifically for this game we developed ASR technology that calculated confidence measures at both sentence and word level (van Doremalen et al., 2010), which were used in the following two stages, respectively. (1) Stage 1 verified whether the user actually attempted to speak the target utterance. This is to prevent the system from providing erroneous feedback on the displayed sentence. (2) Stage 2 detected mispronunciations at word level. If a word confidence measure was lower than a threshold, it was coloured red. Feedback was only provided on nouns and verbs (they provide the largest part of information). Prepositions, articles and pronouns were always coloured green. Tests with this procedure using artificially generated data showed that feedback was sufficiently reliable for our application.

## MATERIALS AND METHODS

### Design

**FIGURE 2:**
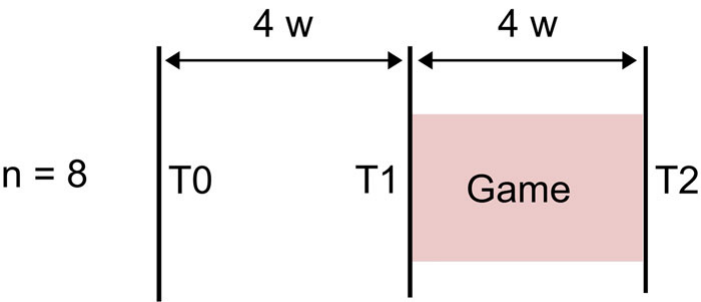
The repeated measures design used to study the effects of the game intervention (w = weeks). T2 and T3 were the speech pre‐ and post‐tests [Colour figure can be viewed at wileyonlinelibrary.com]

A single‐group repeated measures design was used to study the effects of our game‐based speech training intervention (Figure 2). Since this study was of an exploratory nature and it was quite difficult to include matched participants in a control group due to their heterogeneous characteristics (Beijer et al., 2014, Ganzeboom et al., 2018), we refrained from a control group. Each participant received a 4‐week intervention using Treasure Hunters 2. Repeated measures (i.e., speech recordings) were carried out before and after the intervention, at T2 and T3. Speech recordings were also made at T1, 4 weeks before T2. In this way, the effects of the intervention were compared with an equally lengthened period without intervention. It was also used to check the (recording and playing) conditions for the intervention at the participants’ home environment, minimizing and removing any noise sources (e.g., traffic, humming lights, ticking clocks, etc.). At T3 participants also completed a user satisfaction questionnaire and a paired comparisons preference task (hereafter denoted as ‘preference task’).

### Participants

Patients with dysarthria due to PD were recruited via speech therapists, patient internet fora and Facebook groups. The patients had completed their latest face‐to‐face speech training at least two months before T1. Exclusion criteria were aphasia, reported severe cognitive problems or other disabilities that would hamper 15 min training sessions with the game. From the 15 participants who were found willing to participate, seven had to be excluded because of the aforementioned reasons.

Eight participants, five male and three female, all with dysarthria due to PD, completed the intervention and were included in our study. Table 1 provides the demographic data. All participants used Levodopa medication to improve motor functioning, except for participants 5 and 6 who used no medication. Participant 3 had a hearing aid. Participant 5 has received a deep brain stimulation (DBS) system implant in the past and started experiencing difficulties with speaking afterwards. All participants using Levodopa were recorded in the ON condition at all measurement times. Participant 5 was equally recorded in the ON condition with the DBS implant turned on.

**TABLE 1:** Participant self‐reported characteristics

**Participant**	**Gender**	**Age (years)**	**Time since diagnosis (years)**	**Mobility limitations**	**Perceived impact on daily communication**	**Experience with tablets**
p1	Male	73	4.5	Little	Large	A lot
p2	Male	56	8.0	None	Large	None
p3	Male	60	4.5	None	Large	Considerable
p4	Male	63	5.0	None	Large	A lot
p5	Female	53	9.0	None	Large	A lot
p6	Male	75	2.0	Severe	Large	Considerable
p7	Female	67	3.0	None	Large	Considerable
p8	Female	62	3.0	Little	Large	A lot

All participants experience dysarthria due to PD. Levodopa medication is also used by all, except for participants 5 and 6. Options for the participants’ own perceived limitations on mobility around the home environment were ‘none’, ‘little’ or ‘severe’. Options for the perceived impact on their daily communication were ‘none’, ‘little’ and ‘large’. Options for the assessment of computer skills were ‘a lot’, ‘considerable’, ‘little’, ‘hardly’ and ‘none’.

### Speech training intervention

In line with the PLVT protocol (De Swart et al., 2003), the speech intervention consisted of 15‐min practice sessions, four times per week for 4 consecutive weeks.

All participants played the game on the same model tablet (Apple iPad Air) on a desk stand, using a headset (Sennheiser PC 3 Chat) for voice communication. They played the game together with a co‐player through the internet. Co‐players were recruited university student assistants who studied speech and language therapy/pathology. They were instructed to act as a cooperative co‐player and to encourage the participant to speak and not to give any feedback on participants' pronunciation. They were only allowed to ask for clarification as in regular conversation (e.g., ‘Could you repeat that?’). The student assistants were also instructed on how to explain the game to the participants and how to set it up in patients’ home environments.

As shown in Figure 2, participants’ speech was recorded in their home at three points in time (T1–T3). Before the recordings at T1 the internet connection was tested, and a low noise location was selected. The recordings at T2 and T3 served as pre‐ and post‐test for the intervention. At T2 the co‐player explained and practised the game with the participant.

### Measurement instruments

#### Speech materials

In order to measure the development of participants’ speech intelligibility, speech recordings were made of the same sentences and texts. During the three recording sessions, participants’ speech was recorded while they read sentences and texts aloud. The same sentences and texts were read at all points in time. The materials to be read aloud were selected to facilitate the measurement of effects the intervention had on the intelligibility of daily speech. Those speech materials should also challenge participants in the affected speech dimensions (i.e., loudness and articulatory precision). Our selection comprised 30 sentences containing a word with /p/, /t/ or /k/ as the initial sounds (Beijer et al., 2014); we call the associated utterances /p/, /t/ and /k/ sentences. These sentences sufficiently challenged patients’ maintenance of loudness levels and pitch as well as their articulatory precision. Additionally, the short story ‘Papa en Marloes’ (Van de Weijer & Slis, 1991) was included in our selection because of its focus on oral and nasal speech sounds. Lastly, the ‘Apple pie recipe text’ (Ganzeboom et al., 2018) was included to have a more engaging text that stimulates the realization of functional speech. To that end, participants were asked to imagine reading a recipe to a friend who was ‘busy baking the pie at the front of the kitchen while the participants were at the back’. The distance they had to cover would naturally encourage participants to speak loudly.

During the speech assessments, the recording of the speech stimuli was restricted to a single attempt. However, additional recording attempts were made if any reading errors, stutters or restarts occurred as these would negatively bias later judgements of intelligibility. To limit their occurrence, participants read the text silently before starting the recording. Participants' speech was recorded at a sampling rate of 44.1 kHz and 16‐bit Pulse Code Modulation (PCM) resolution.

#### Measuring the effect on speech intelligibility

The speech samples recorded in the speech assessment sessions were judged on intelligibility by inexperienced listeners in an adapted paired comparisons judgement task. We chose inexperienced listeners, because they represented individuals from daily life who are unfamiliar with participants and dysarthric speech in general. For each participant, 10 inexperienced listeners—university students—were asked to judge the intelligibility of the speech samples recorded at all three time points (i.e., T1–T3) as compared with the intelligibility of a neurologically healthy reference speaker. We chose to use neurologically intact speech as ‘anchor’ in the paired sample in order to facilitate judging the extent to which participants’ speech deviates from typical speech. We used a female reference speaker for the female participants and a male reference speaker for the male participants, both with ages similar to those of the participants (67 and 69 years). The reference speakers recorded the same speech materials as the participants, using the same model laptop and headset as the participants.

In the paired comparisons judgement task, the speech sample of the participant was played first and that of the reference speaker second. Listeners were then asked to what extent the first sample was less intelligible than the second. They assigned their ratings using a 7‐point scale from –5 (the first sample was considerably less intelligible than the second) to 1. On this scale, 0 represented both samples being equally intelligible and 1 to provide the possibility to indicate that the first sample was more intelligible than the second. A negative scale was used as we believed this would be more intuitive to indicate poorer intelligibility. Similarly, a positive integer could be used to indicate better intelligibility. Various pilot rating sessions showed no problems using this scale.

Speech samples for the judgement task were selected from the recorded speech materials. The recordings from the /p/, /t/ and /k/ sentences were used without changes. To increase the number of judgements for our analysis of efficacy, the recordings from the text fragments were cut at each sentence boundary, creating individual recordings that were rated separately.

Recordings containing unrecoverable reading errors and (background) noise were excluded from the judgement. To further prevent bias in judgements, a selection of recordings was made that was balanced in length (expressed in number of phonemes), occurrence of non‐frequent words (lemma frequency < 10 per million), the number of occurrences of the /p/, /t/ and /k/ sounds and the number of consonant clusters. The final set contained 42 speech samples. All speech samples were normalized to an average intensity of 68 dB(A) (calibrated to an artificial ear; Brüel Kjaer 2610).

In total, each listener was asked to judge 126 speech samples of one, randomly assigned, participant (42 pairs per time point). The samples were digitally provided to the listeners in an OpenSesame experiment (Mathôt et al., 2012). The order in which the different pairs were presented was randomized.

#### User satisfaction

Participants filled in a user satisfaction questionnaire at time point T3, after finishing the intervention (see Supporting information A in the additional supporting information). Google Forms was used to make this questionnaire available online. Five items of the questionnaire were based on previous research (Beijer, 2012: ch. 8; Ganzeboom et al., 2018). Four items asked the users about their satisfaction with the interface, ease of use, attractiveness and the overall system. Each of these were rated on a 10‐point scale, ranging from 1 (extremely unsatisfied) to 10 (extremely satisfied). Ratings below 6 indicated an insufficient level of satisfaction. Dutch people are familiar with this scale since it is commonly used in the Dutch school system (NUFFIC, 2022). The fifth question was open ended and asked how users generally experienced playing the game.

At time points T1 and T2 it became apparent that most participants would find it difficult to fill in the questionnaire online due to physical limitations or fatigue. We therefore decided that the student assistants that made the speech recordings should read the questions aloud and make an audio recording of users’ responses. Student assistants were instructed to limit the text they read aloud to the question on the questionnaire and, if necessary, the accompanying explanatory sentence.

#### User appreciation of feedback

Five open‐ended questions were added to the user satisfaction questionnaire in order to measure the appreciation of the feedback on loudness, pitch and pronunciation (see Supporting information A in the additional supporting information). The questions regarding loudness and pitch assessed whether participants noticed the feedback, how they experienced its precision, and if they could act on it (i.e., change the use of their voice in accordance with the feedback). Questions regarding pronunciation probed users’ general experience of the feedback and to what extent they disagreed with the provided feedback. Screenshots of the game accompanied these questions and were referred to in the question's text. The responses to these questions were audio recorded in the same session as the questions on user satisfaction.

#### User preference

**FIGURE 3:**
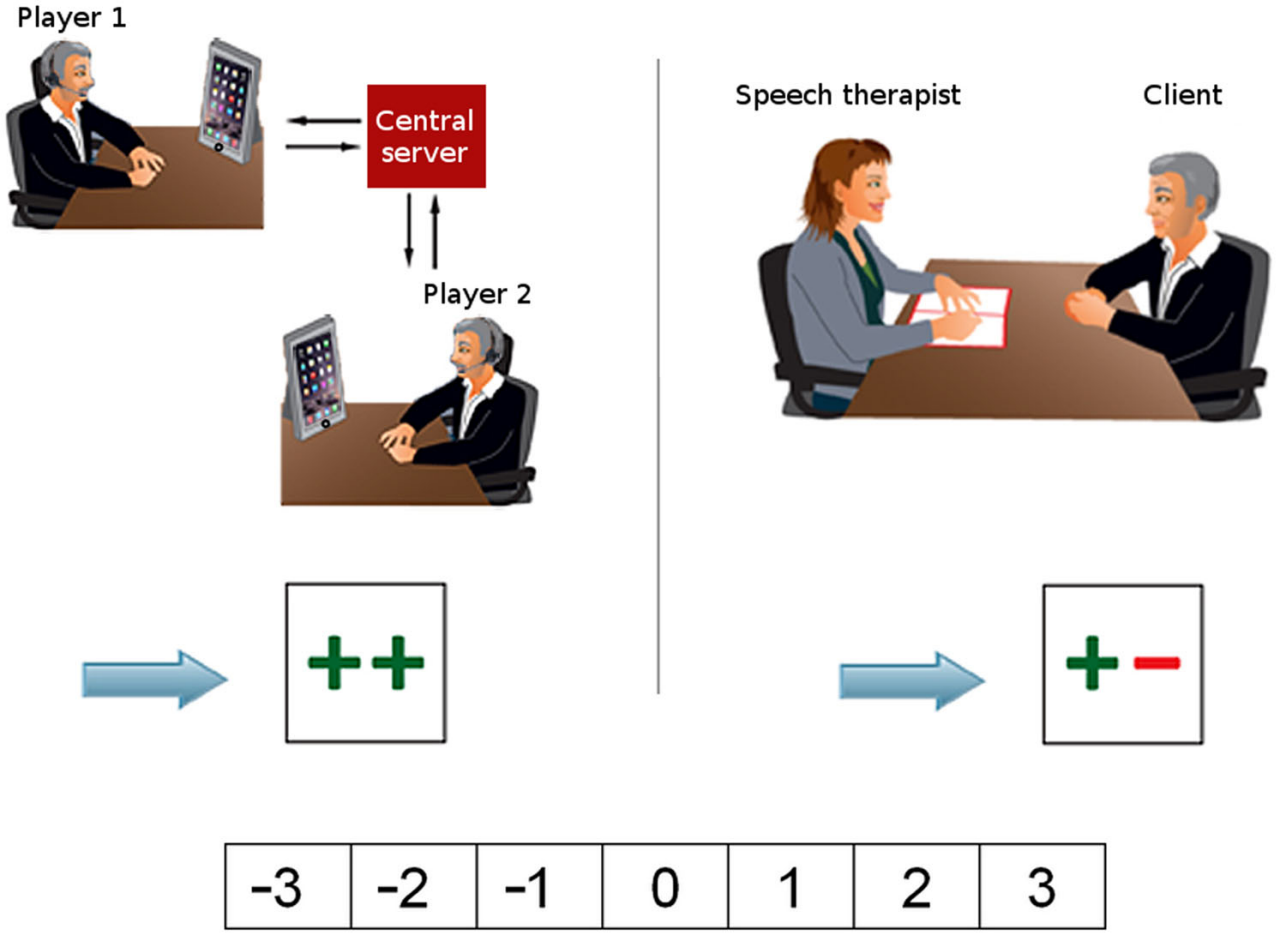
Example of a scenario in the preference task. The participant had to choose between a game‐based intervention with strong hypothetical improvement (++) and a face‐to‐face‐based intervention with slight hypothetical improvement (+–). Scenarios were rated from –3 (a strong preference for the left option) to 3 (a strong preference for the right option) [Colour figure can be viewed at wileyonlinelibrary.com]

**FIGURE 4:**
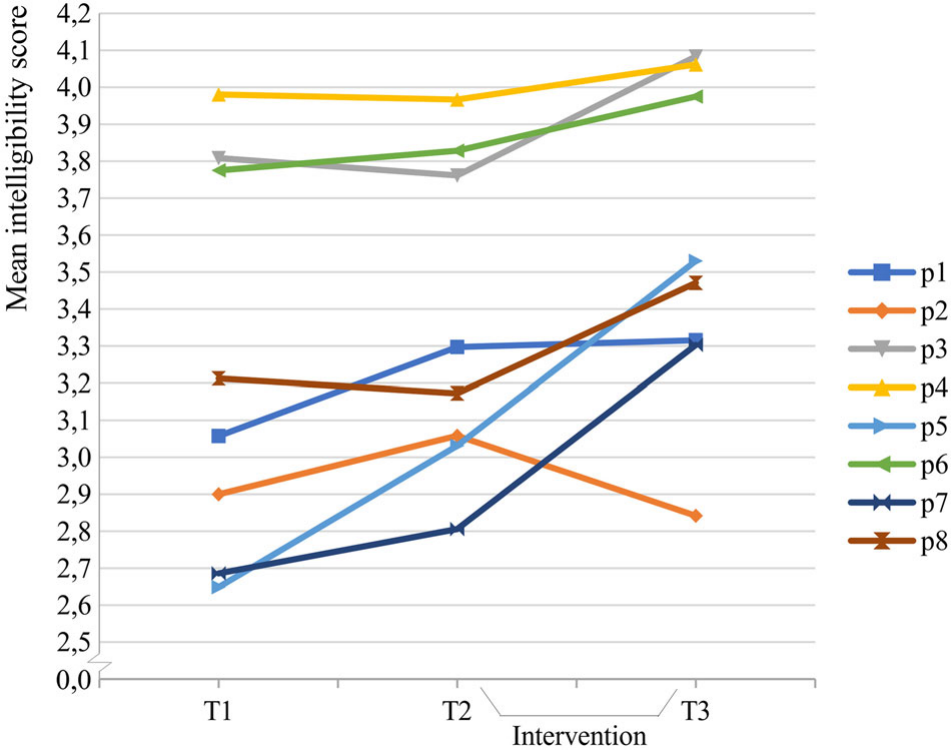
Estimated marginal means of intelligibility scores per speaker per time point. [Colour figure can be viewed at wileyonlinelibrary.com] *Note*: Ratings are on a scale of 0–5. The graph is zoomed in on the *y*‐axis

In the preference task, based on Beijer (2012: ch. 8), we explored users’ preference for game‐based speech training relative to face‐to‐face training in scenarios where the two training methods lead to different hypothesized levels of speech improvement. The participants were asked to choose between game‐based speech training (using a two‐player tablet game) and traditional face‐to‐face speech training in four of such scenarios. Hypothesized levels of speech improvement were attached to the two interventions and were either a ‘slight improvement’ (+–) or a ‘strong improvement’ (++). An example of a scenario is shown in Figure 3 where the participant had to choose between a game‐based intervention with strong hypothetical improvement and a face‐to‐face‐based intervention with slight hypothetical improvement. [Corrections made on 5 April 2022, after first online publication: In this version, Figure 4 citation has been removed from the previous sentence.] Each scenario was rated on a scale from –3 (strong preference for the left option) to 3 (strong preference for the right option). The hypothetical scenarios were presented visually to participants through the E‐Prime 2 software (E‐Prime 2, 2012) and were orally explained by the experimenter.

### Data analysis

To facilitate analyses, listeners’ judgments were converted to a positive scale, 0 (the participant's sample was considerably less intelligible than that of the reference speaker) to 5 (both samples being equally intelligible). A higher score thus represents higher intelligibility. The original 1 score, indicating that the participant's sample was more intelligible than that of the reference speaker, was not often used (3.5% of total judgements). After inspection, we were confident that these were due to small regional variations in pronouncing individual speech sounds or recording conditions (i.e., breathing in the microphone and background noises). For that reason, the speech samples corresponding to these judgements were considered as equally intelligible and merged with the category ‘equally intelligible’ (5 in our converted scale).

From the 42 sentences recorded per speaker for every time point, 24 had usable recordings available for all three measurement times. The other sentence recordings could not be used because recordings of one or multiple measurement times contained an unrecoverable event (e.g., speech error, breathing in the microphone, environmental noise, etc.) that could bias listeners’ judgements. Consequently, judgements of 24 speech samples were used for every measurement time by 10 listeners, totalling 720 judgements per speaker.

For each of the eight speakers and for each time point (T1–T3) reliability of the listeners’ judgements was assessed by the intraclass correlation coefficient, ICC(2,k). This version of the ICC family assumes that both raters and objects are random factors (Rietveld, 2021). A total of 21 of the 24 estimates of reliability (eight speakers three time points) were significant (*p* < 0.05), while three were not. The data of these three were further investigated on irregularities (i.e., possible errors in judgments, scale conversion errors and abnormalities in judged recordings), but none was found. The mean value of the 24 estimates of reliability was 0.678.

The results of the listening experiment were analysed using the *lmer* function of the R package *lme4* (Bates et al., 2015) for linear mixed effects modelling. The fixed factors in the model were Speaker and Time, and their interaction; the random factor was Utterance (random intercept). The effect sizes were measured with the index *R* ^2^_Partial_ (the Kenward–Roger approach, provided by the R‐procedure *r2glmm* ^1^). To further inspect differences between time points, we carried out paired comparisons, using the R‐procedure *emmeans*.^2^

For the user satisfaction data, the recordings of the participants were listened to by the first author and the ratings they gave were noted in a table. Descriptive statistics were calculated, consecutively. The responses to the open question about how participants generally experienced Treasure Hunters 2 were summarized.

Equal to the user satisfaction data, the responses to the open questions about the appreciation of feedback on pitch, loudness, and pronunciation were also listened to and summarized.

For the user preference data, descriptive statistics were calculated.

## RESULTS

The intervention included 16 speech training sessions with the game. For health‐related reasons and technical issues, not all participants completed all sessions: three participants completed all 16 sessions, three participants completed 15, one completed 14 sessions and one completed 13 sessions.

### The effect of the intervention on speech intelligibility

Speaker, Time and their interaction were found to be significant factors (*p* < 0.01). The results of the fit by the R‐procedure restricted maximum likelihood (REML) are given in Table 2.

**TABLE 2:** Results of the REML procedure on the intelligibility scores

**Factor**	***F***	**df1, df2**	***p*‐value**	***R* ^2^_Partial_**
Speaker	49.77	7, 529	< 0.01	0.397
Time	9.655	2, 529	< 0.01	0.070
Speaker × Time	8.528	14, 529	< 0.01	0.062

The marginal means of each speaker at each time point are displayed in Figure 4.

Table 3 shows the results of our paired comparisons of intelligibility ratings carried out with the emmeans R‐procedure.

Whereas the difference of the scores obtained at T1 and T2 is not significant, that of the scores obtained at T2 (start of the treatment) and T3 was.

**TABLE 3:** Results of the paired comparisons of intelligibility ratings carried out with the emmeans R‐procedure

**Contrast**	**Estimate**	**SE**	**d.f**.	***t*‐ratio**	***p*‐value**
T1–T2	0.104	0.0504	529	2.065	0.0982
T1–T3	0.311	0.0504	529	6.184	< 0.0001
T2–T3	0.207	0.0504	529	4.119	0.0001

### User satisfaction

The participants’ user satisfaction ratings for the game are displayed in Table 4. None of the participants rated the game as insufficient.

**TABLE 4:** User satisfaction ratings per participant

**Participant**	**Interface**	**Ease**	**Attractiveness**	**Overall**
p1	8	8	8	9
p2	6	5	8	7
p3	7	7	7	7
p4	7	7	8	7
p5	9	9	8	9
p6	7	7	6	6
p7	9	9	9	7
p8	7	6	8	6
Average per dimension	7.50	7.25	7.75	7.25

Ratings on four dimensions: satisfaction with the Interface, Ease of use, game's Attractiveness and Overall rating of the gaming experience. A 10‐point scale was used, in which 6 is considered ‘sufficient’.

All participants but for one provided comments on their overall experience playing the game. They stated that they liked the game and assumed that it helped train their speech.

## User appreciation of feedback

All participants except one stated that they frequently noticed the feedback throughout the game. For the question on the quality of the feedback, the answers were mixed. All but one participant answered positively to the question whether they could use the feedback and attempted to speak louder and lower.

Feedback on pronunciation was described as a positive experience by most participants as well. Five participants always agreed with the given feedback. Two sometimes disagreed and thought that the system should have detected a mispronunciation when it did not. One participant sometimes disagreed and was puzzled by why an initial pronunciation was not correct and a second was.

## User preference

Table 5 presents the participants’ ratings obtained in the preference task in which they were asked to choose between game‐based and face‐to‐face speech training in four hypothetical situations (positive scores indicate a preference for game‐based speech training). The available four comparisons per participant provided patterns in the criteria used in the preferences: preference for either game or face to face, possibly ‘modulated’ by the hypothetical outcome of the therapy. When the preferences are converted into binary scores, we see that four participants always prefer gaming, unless the outcome of the face‐to‐face therapy is strongly positive and that of the gaming only slightly positive; for two participants it is the other way round: face to face, unless the outcome of the face‐to‐face therapy is slightly positive and that of the gaming strongly positive, whereas two other participants show no consistent patterns in their preferences. These patterns are only based on observations with eight participants. Nevertheless, it makes clear which patterns can be expected, on the basis of which criteria: gaming versus face to face, and the modulation of these preferences by the quality of the outcomes.

**TABLE 5:** Preference ratings per participant comparing game‐based speech training (G) with face‐to‐face speech training with a therapist (F). The hypothetical outcomes of the therapy are marked as strong (++) or slight (+–) improvement

	**Preference per scenario**				**Average preference game**
					**versus face to face**
**Participant**	**G++ versus F++**	**G+– versus F+–**	**G++ versus F+–**	**G+– versus F++**	
p1	2	2	3	–2	1.25
p2	–3	–3	3	–3	–1.50
p3	1	1	2	–2	0.50
p4	3	3	3	–2	1.75
p5	–3	–2	2	–3	–1.50
p6	3	–2	3	–3	0.25
p7	3	3	3	–3	1.50
p8	2	–2	2	–3	–0.25

*Note*: Ratings range from –3 to 3, with positive values indicating a preference for game‐based speech training over face‐to‐face speech training with a therapist.

## DISCUSSION

Since there is an urgent need for independent, prolonged speech training in neurological patients’ home environment, this study explored the potentials of game‐based speech training for dysarthric speakers with PD.

Pre‐post‐measurements indicated a statistically significant improved speech intelligibility after the intervention (T2–T3), whereas no significant changes in speech intelligibility were observed over a 4‐week period of no training prior to the intervention (T1–T2).

Moreover, the dysarthric speakers graded their satisfaction regarding the interface, the ease of use, the attractiveness of the game and their satisfaction overall with Treasure Hunters 2 as a game for speech training on average with 7.37 on a 10‐point scale (no outliers). Although these preliminary results are promising, we should note that despite the statistically significant improvement, the clinical differences are relatively small. A contributing factor may have been the limited duration of our treatment. Our four weeks of treatment is based on intensive treatments in previous research as described in the Materials and Methods section. One could reason that additional weeks of treatment may improve speech intelligibility even more.

Our preliminary results do not show a consistent preference for game‐based speech or traditional face‐to‐face speech training. Regardless of preferred type of training, users’ decisions on the preferred scenario seem to be affected by the hypothesized effects on speech intelligibility. That is, our results indicate that the decisive factor seems to be the beneficial effects on speech, rather than the type of speech training. Similar results were observed in previous research (Beijer, 2012: ch. 8; Ganzeboom et al., 2018). This also indicates no direct preference for traditional face‐to‐face training. Consequently, dysarthric speakers of the age group included in our study appear to positively receive game‐based types of speech training as an alternative. The trade‐off between type and effect of training calls for research into determinants for effective game‐based training. From this perspective, our exploration of dysarthric speakers’ appreciation of the type of feedback on loudness, pitch and pronunciation is relevant. That is, we included several types of feedback and explored how dysarthric speakers used and interpreted them. Overall, the dysarthric speakers in our study noticed the feedback on loudness and pitch when it was given, agreed with it most of the time and were able to adjust their speech as a result.

Generally, the types of feedback that were used seem to be well received in our study, especially the combination of implicit and explicit feedback for loudness. That is in line with Bakker et al. (2018) considerations for effective feedback. Reflecting on their work, we believe that a combination of implicit and explicit feedback can reinforce each other such that the explicit feedback makes the implicit feedback easier to understand.

In our exploration of feedback on pronunciation we employed ASR technology. As also pointed out by Bakker et al. (2018), it is important to consider the feasibility of providing reliable speech feedback. Our tests with artificially generated data made us confident that it would. However, we also know that artificially generated data does not reflect real data in every way. In our study, most participants had a positive experience and did not disagree with the provided feedback. Two participants described that the game sometimes fails to recognize mispronunciations. In ASR terms, the game sometimes falsely accepts mispronunciations. False rejects were not reported by the participants. In language learning literature false rejects are usually regarded as more detrimental to the process of language learning than false accepts (Doremalen et al., 2013). We assume that the same applies to speech training.

The duration of our speech training (i.e., 4 weeks) might be considered limited and perhaps contributed to an incomplete view of the effects of game‐based training. Furthermore, no statements can be made about long‐term effects of our treatment.

From a technological point of view we note that the calibration of the thresholds used for sentence and word verification was based on artificially generated data.

Future research should entail the calibration of these thresholds on real data. The data that was collected during this study can be used for that purpose. Furthermore, future studies should include a larger number of participants to enable more robust conclusions about the effects of game‐based speech training. Also, the long‐term effects of our treatment should be the subject of future study. Previous research showed that patients with dysarthria due to stroke also benefit from intensified and prolonged training (Wenke et al., 2008). For that reason, stroke patients should be included in future efficacy studies into serious games for speech training.

## CONCLUSIONS

In this study the effects of an improved game‐based speech intervention were compared with a period without intervention. We observed no significant differences between the pre‐ and post‐measurements of the period without intervention, but did find a significant effect of our intervention. This finding supports game‐based speech training as a positive modality to improve the overall speech intelligibility of individuals with dysarthria due to PD.

In general, the dysarthric patients are satisfied with game‐based speech training. Except for one outlier, all ratings were 6 or higher, and the total average is 7.25 on a 1–10 scale.

All but one participant had noticed the feedback on loudness, pitch and pronunciation, and they were positive about it. They also felt they could use the feedback provided by the system to improve the quality of their speech.

Regarding their preference for game‐based versus face‐to‐face training, users do not prefer game‐based speech training above face‐to‐face training. Most users have a preference for the type of speech training which provides the most hypothetical improvement.

To summarize, patients are positive about game‐based speech therapy with instantaneous feedback, under the condition that a significant improvement is assured. However, these preliminary results should be interpreted with care. As in previous studies, differences between patients were observed. We can thus conclude that the game should be carefully tailored to each patient. Furthermore, for an optimal effect and user satisfaction, it should preferably not be used in isolation but in combination with face‐to‐face training. This way, the strengths of both types of training can be used to improve speech intelligibility in patients with PD.

## CONFLICT OF INTEREST

The authors declare no conflict of interest in the work reported here.

## FUNDING

This research was funded by the NWO research grant with Ref. no. 314‐99‐101 (CHASING).

## Supporting information



Supplementary material

## Data Availability

Research data are available on the condition that if the data are used in any publication, presentation or other form of communication, the authors are acknowledged and this paper is cited. The data have been made available through the Radboud Data Repository at https://doi.org/10.34973/h19s‐c122. Ethics approval was not required as stated by the Radboud University Medical Centre Research Ethics Committee (2015‐1707). Patients were recruited via speech pathologists, patient internet fora and Facebook groups. They were given information about our research and assured they could withdraw at any time. They signed consent forms for participation and the collection of speech recordings. These were uploaded via the web using a secure connection and stored at a secure locationvv where only the first and last authors had access to. All the patient data were anonymised.

## NOTES

1 See https://cran.r‐project.org/package=r2glmm for more details on the r2glmm package.

2 See https://cran.r‐project.org/package=emmeans for more details on the emmeans package.

## References

[jlcd12722-bib-0001] E‐PRIME 2 (Version 2.0.8.90) [Computer software], 2012, Pittsburgh, PA: Psychology Software Tools.

[jlcd12722-bib-0002] Bakker, M. , Beijer, L. & Rietveld, T. (2018) Considerations on effective feedback in computerized speech training for dysarthric speakers. Telemedicine and e‐Health, 25(5), 351–358. 10.1089/tmj.2018.0050. 30074851

[jlcd12722-bib-0003] Bates, D. , Mächler, M. , Bolker, B. & Walker, S. (2015) Fitting linear mixed‐effects models using lme4. Journal of Statistical Software, 67(1), 1–48. DOI:10.18637/jss.v067.i01.

[jlcd12722-bib-0004] Beijer, L.J. (2012) E‐learning based speech therapy (EST): Exploring the potentials of E‐health for dysarthric speakers, PhD thesis, Radboud Universiteit, Nijmegen, viewed 15 February 2021, https://hdl.handle.net/2066/101662

[jlcd12722-bib-0005] Beijer, L.J. , Rietveld, A.C. , Ruiter, M.B. & Geurts, A.C. (2014) Preparing an E‐learning‐based Speech Therapy (EST) efficacy study: identifying suitable outcome measures to detect within‐subject changes of speech intelligibility in dysarthric speakers. Clinical Linguistics and Phonetics, 28(12), 927–950. 25025268 10.3109/02699206.2014.936627

[jlcd12722-bib-0006] De Bodt, M.S. , Hernández‐Diáz Huici, M.E. & Van De Heyning, P.H. (2002) Intelligibility as a linear combination of dimensions in dysarthric speech. Journal of Communication Disorders, 35(3), 283–292. 12064788 10.1016/s0021-9924(02)00065-5

[jlcd12722-bib-0007] de Swart B.J.M. , Willemse, S.C. , Maassen, B.A.M. & Horstink, M.W.I.M. (2003) Improvement of voicing in patients with Parkinson's disease by speech therapy. Neurology, 60(3), 498–500. 12578936 10.1212/01.wnl.0000044480.95458.56

[jlcd12722-bib-0008] Ganzeboom, M. , Yilmaz, E. , Cucchiarini, C. & Strik, H. (2016) On the development of an ASR‐based multimedia game for speech therapy: preliminary results. Proceedings of the 2016 ACM Workshop on Multimedia for Personal Health and Health Care (ACM), pp. 3–8.

[jlcd12722-bib-0009] Ganzeboom, M.S. , Bakker, M. , Beijer, L.J. , Rietveld, A.C.M. & Strik, H. , 2018, Speech training for neurological patients using a serious game. British Journal of Educational Technology, 49(4), 761–774. 10.1111/bjet.12640.

[jlcd12722-bib-0010] Kalf, H. , De Swart, B. , Bonnier‐Baars, M. , Kanters, J. , Hofman, M. , Kocken, J. et al. (2011) Guidelines for speech–language therapy in Parkinson's disease. Nijmegen, the Netherlands /Miami (FL), U.S.A: ParkinsonNet/NPF. viewed 15 February 2021, https://www.parkinsonnet.nl/app/uploads/sites/3/2019/11/dutch_slp_guidelines‐final.pdf

[jlcd12722-bib-0011] Kari, T. (2017) Promoting physical activity and fitness with exergames: Updated systematic review of systematic reviews. In: Dubbels, B. (Eds.) Transforming gaming and computer simulation technologies across industries. Hershey, PA: IGI Global, pp. 225–245.

[jlcd12722-bib-0012] Laamarti, F. , Eid, M. & El Saddik, A. (2014) An Overview of serious games. International Journal of Computer Games Technology, 2014, 358152. 10.1155/2014/358152.

[jlcd12722-bib-0013] Lewis, Z.H. , Swartz, M.C. & Lyons, E. J. (2016) What's the point?: a review of reward systems implemented in gamification interventions. Games for Health Journal, 5(2), 93–99. 26812253 10.1089/g4h.2015.0078

[jlcd12722-bib-0014] Mathôt, S. , Schreij, D. & Theeuwes, J. (2012) OpenSesame: an open‐source, graphical experiment builder for the social sciences. Behavior Research Methods, 44(2), 314–324. 22083660 10.3758/s13428-011-0168-7 PMC3356517

[jlcd12722-bib-0015] Mendoza Ramos, V. , Paulyn, C. , Van Den Steen, L. , Hernández‐Diáz Huici, M.E. , De Bodt, M. & Van Nuffelen, G. (2021) Effect of boost articulation therapy (BArT) on intelligibility in adults with dysarthria. International Journal of Language & Communication Disorders. 10.1111/1460-6984.12595 PMC8048921 33484095

[jlcd12722-bib-0016] Moya‐Galé, G. & Levy, E.S. (2019) Parkinson's disease‐associated dysarthria: prevalence, impact and management strategies. Research and Reviews in Parkinsonism, 9, 9–16.

[jlcd12722-bib-0017] NUFFIC . The Dutch organisation for internationalisation and education. Grading systems. [online]. Available at: https://www.nuffic.nl/en/education‐systems/netherlands/grading‐systems [Accessed 7 January 2022].

[jlcd12722-bib-0018] Ramig, L.O. , Sapir, S. , Countryman, S. , Pawlas, A.A. , O'Brien, C. , Hoehn, M. et al. (2001) Intensive voice treatment (LSVT®) for patients with Parkinson's disease: a 2 year follow up. Journal of Neurology, Neurosurgery & Psychiatry, 71(4), 493–498. 11561033 10.1136/jnnp.71.4.493 PMC1763504

[jlcd12722-bib-0019] Rietveld, A.C.M. & Van Heuven, V. J. (2016) Algemene fonetiek [General Phonetics], 4th edition, Bussum, Netherlands: Coutinho.

[jlcd12722-bib-0020] Rietveld, T. (2021) Human measurement techniques for speech and language pathology. London: Routledge.

[jlcd12722-bib-0021] Sapir, S. , Spielman, J.L. , Ramig, L.O. , Story, B.H. & Fox, C. (2007) Effects of intensive voice treatment (the Lee Silverman Voice Treatment [LSVT]) on vowel articulation in dysarthric individuals with idiopathic Parkinson disease: acoustic and perceptual findings. Journal of Speech, Language, and Hearing Research, 50(4), 899–912. 10.1044/1092-4388(2007/064) 17675595

[jlcd12722-bib-0022] Van De Weijer, J. & Slis, I. (1991) Nasaliteitsmeting met de nasometer [Measuring nasality using the nasometer]. Logopedie & Foniatrie, 63, 97–101.

[jlcd12722-bib-0023] van Doremalen J. , Cucchiarini, C. & Strik, H. (2010) Optimizing automatic speech recognition for low‐proficient non‐native speakers. Journal on Audio, Speech, and Music Processing. 10.1155/2010/973954.

[jlcd12722-bib-0024] van Doremalenj, J. , Cucchiarini, C. & Strik, H. (2013) Automatic pronunciation error detection in non‐native speech: the case of vowel errors in Dutch. The Journal of the Acoustical Society of America, 134(2), 1336–1347. 23927130 10.1121/1.4813304

[jlcd12722-bib-0025] Van Vilsteren, C. , Gerritsen, E. , Pols, H. , De Jong, L. , Hogendoorn, P. , Schikan, H. et al. Knowledge and innovation agenda 2020–2023 health and care. [online]. Available: https://www.health‐holland.com/sites/default/files/downloads/Knowledge‐and‐Innovation‐Agenda‐2020‐2023‐health‐and‐care.pdf [Accessed 12 January 2022].

[jlcd12722-bib-0026] Wenke, R.J. , Theodoros, D. & Cornwell, P. (2008) The short‐ and long‐term effectiveness of the LSVT^®^ for dysarthria following TBI and stroke. Brain Injury, 22(4), 339–352. 10.1080/02699050801960987. 18365848

[jlcd12722-bib-0027] Wight, S. & Miller, N. (2015) Lee Silverman Voice Treatment for people with Parkinson's: audit of outcomes in a routine clinic. International Journal of Language & Communication Disorders, 50, 215–225. 10.1111/1460-6984.12132 25469736

[jlcd12722-bib-0028] World Health Organization . (2005) Resolution WHA58.28: eHealth. [Online]. WHA58.28. Available from: http://extranet.who.int/iris/bitstream/10665/20378/1/WHA58_28‐en.pdf?ua=1 [Accessed 7 January 2022].

