# Supplementary material for: A serious game for speech training in dysarthric speakers with Parkinson's disease: Exploring therapeutic efficacy and patient satisfaction
Source: Int J Lang Commun Disord. 2022 Mar 26;57(4):808–21. doi: 10.1111/1460-6984.12722 (PMC9543756; doi:10.1111/1460-6984.12722)
Supplement: Supplementary file 1 — Supplementary material [file JLCD-57-808-s001.docx]

**Supporting information**

**A User satisfaction questionnaire**

**Note: the version below is a translation from the Dutch version used in our research.**

**User satisfaction Treasure Hunters**

The following questions are about your satisfaction with Treasure Hunters, the game on the iPad you trained with in the last couple of weeks.

Answer the questions by clicking on one of the round buttons underneath. The scale goes from 1 to 10, 1 being very unsatisfied and 10 very satisfied.

**Your participant number**
Your test leader can enter this for you.

|  |
| --- |

**How satisfied are you with Treasure Hunters’ user interface?**With this we mean the layout of the screen, use of the buttons, and possible help texts.

|  | 1 | 2 | 3 | 4 | 5 | 6 | 7 | 8 | 9 | 10 |  |
| --- | --- | --- | --- | --- | --- | --- | --- | --- | --- | --- | --- |
| Extremely unsatisfied | 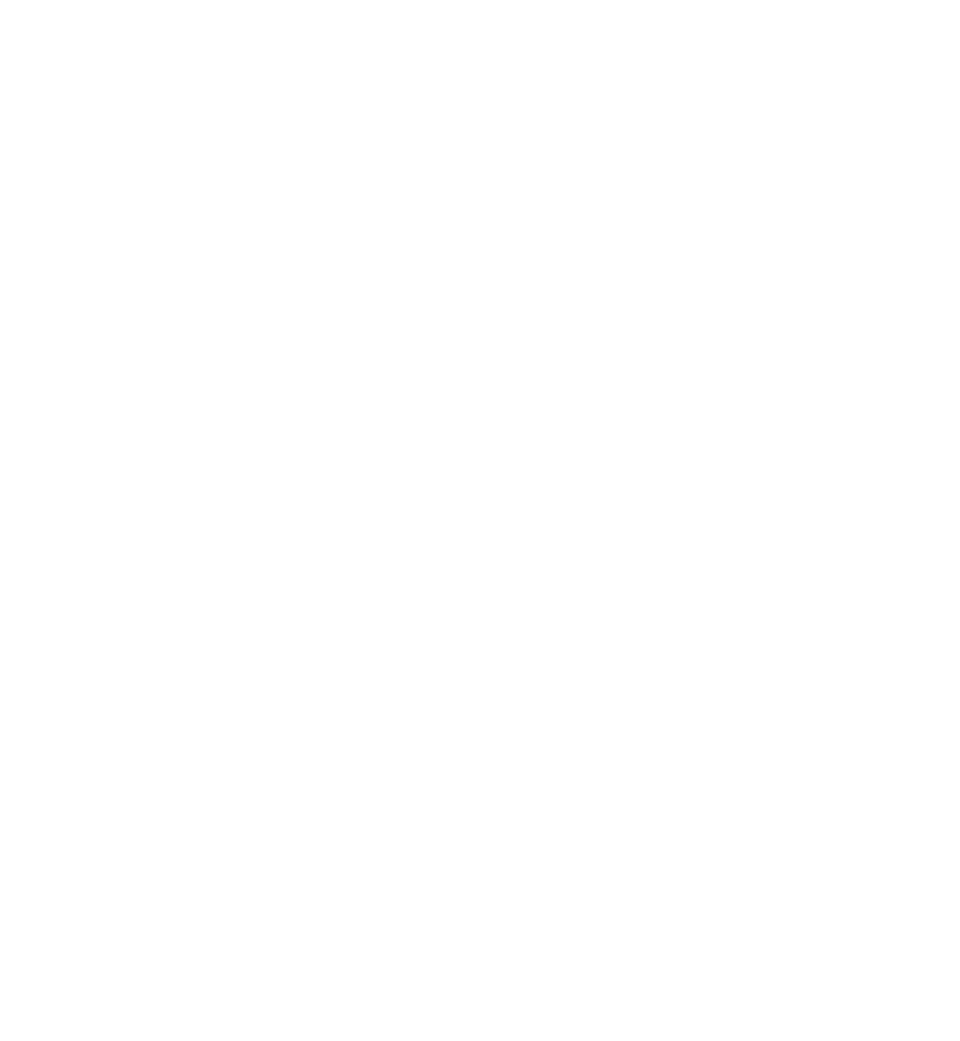 | 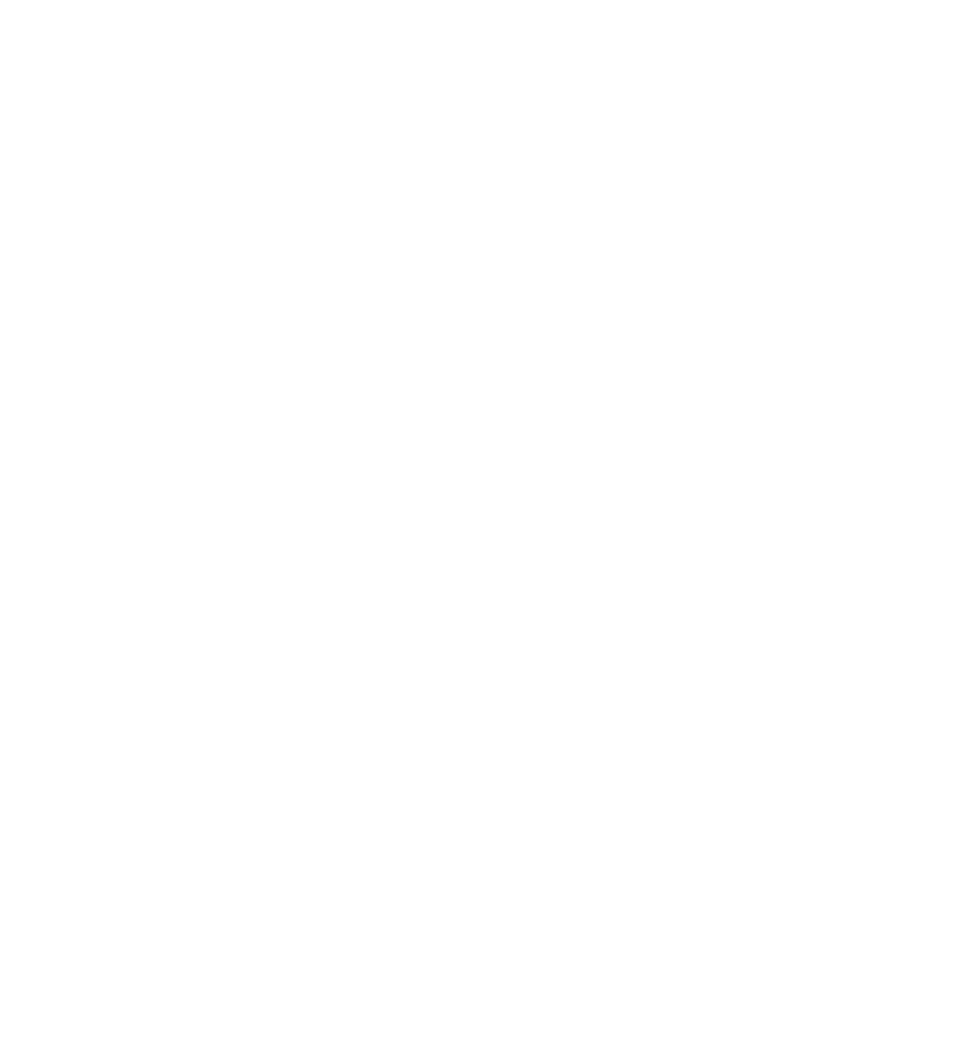 | 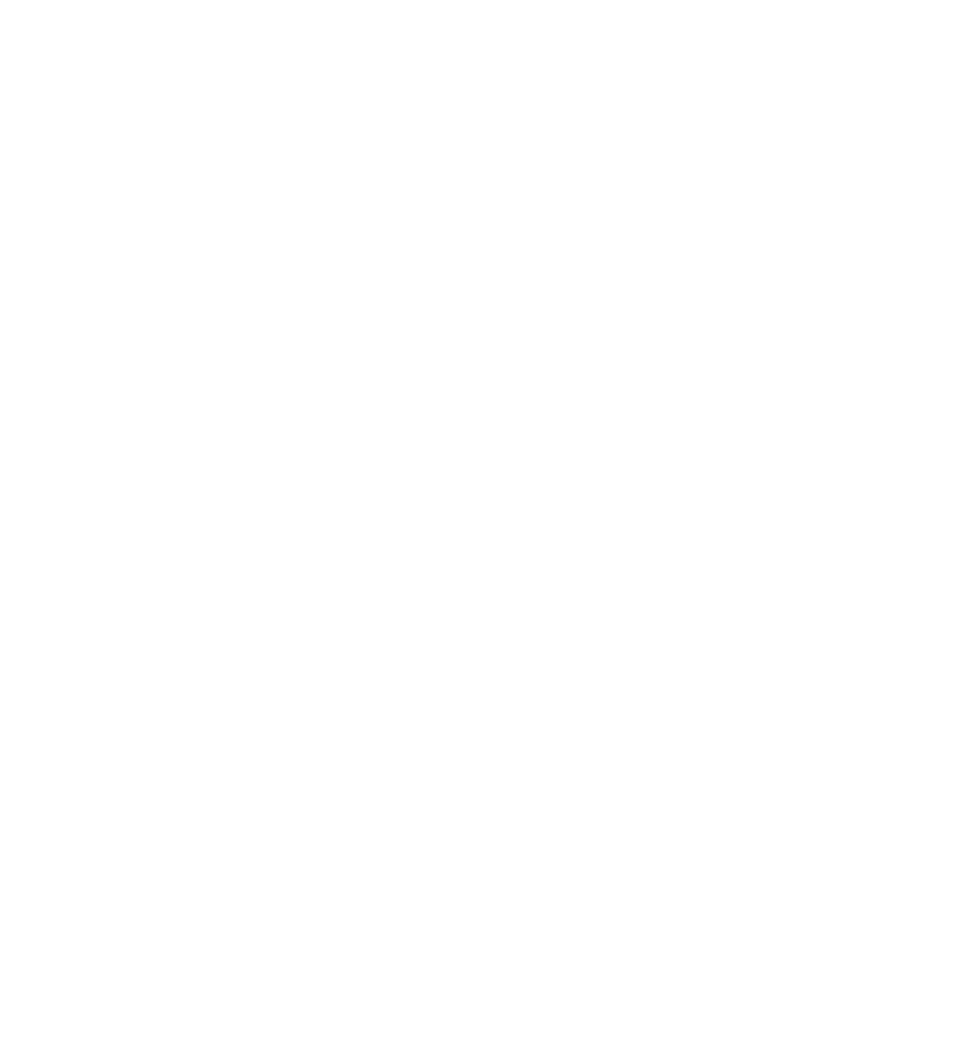 | 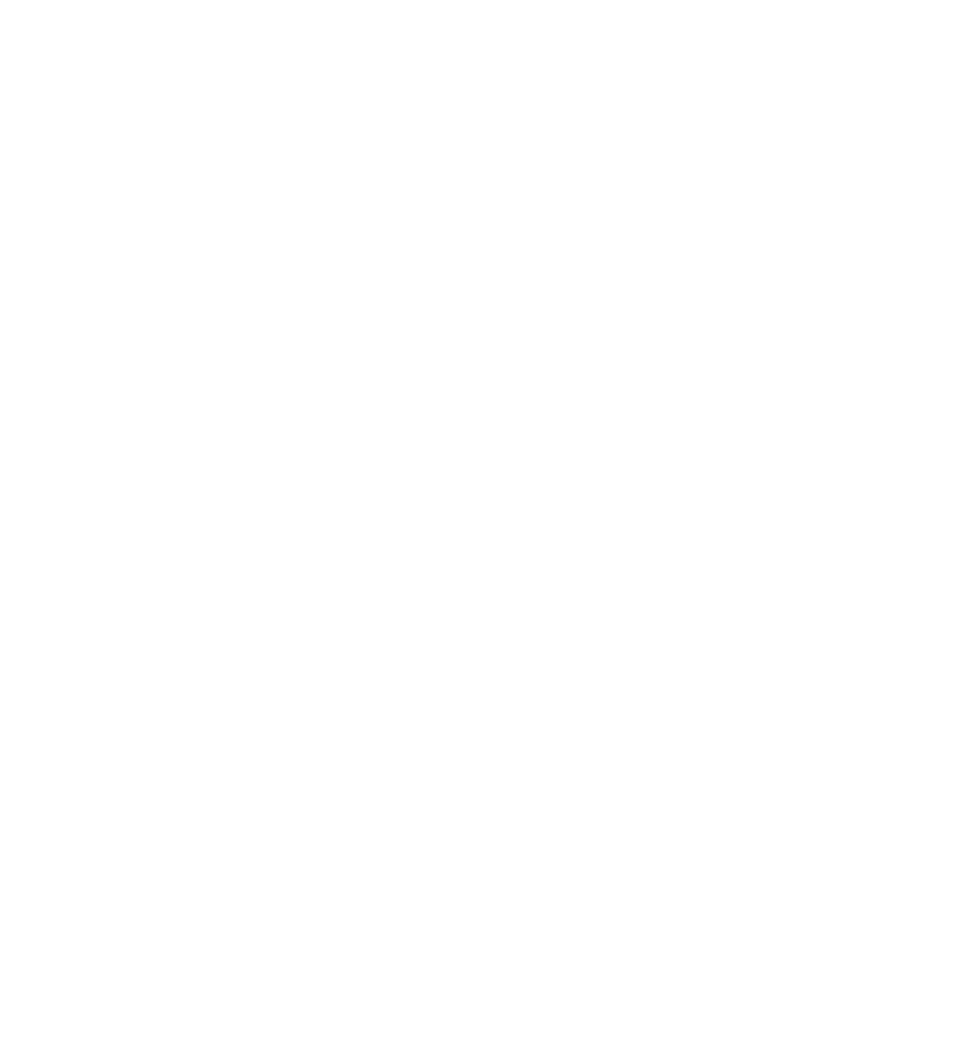 | 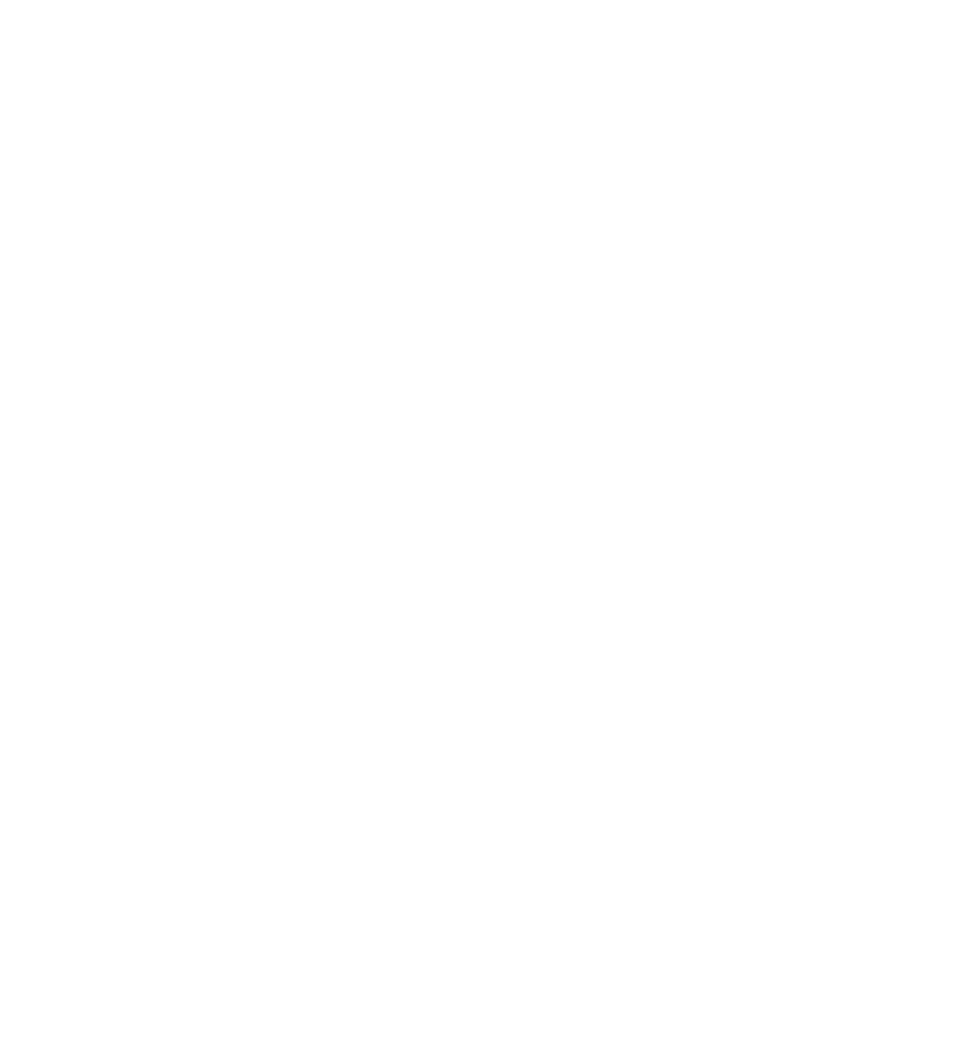 | 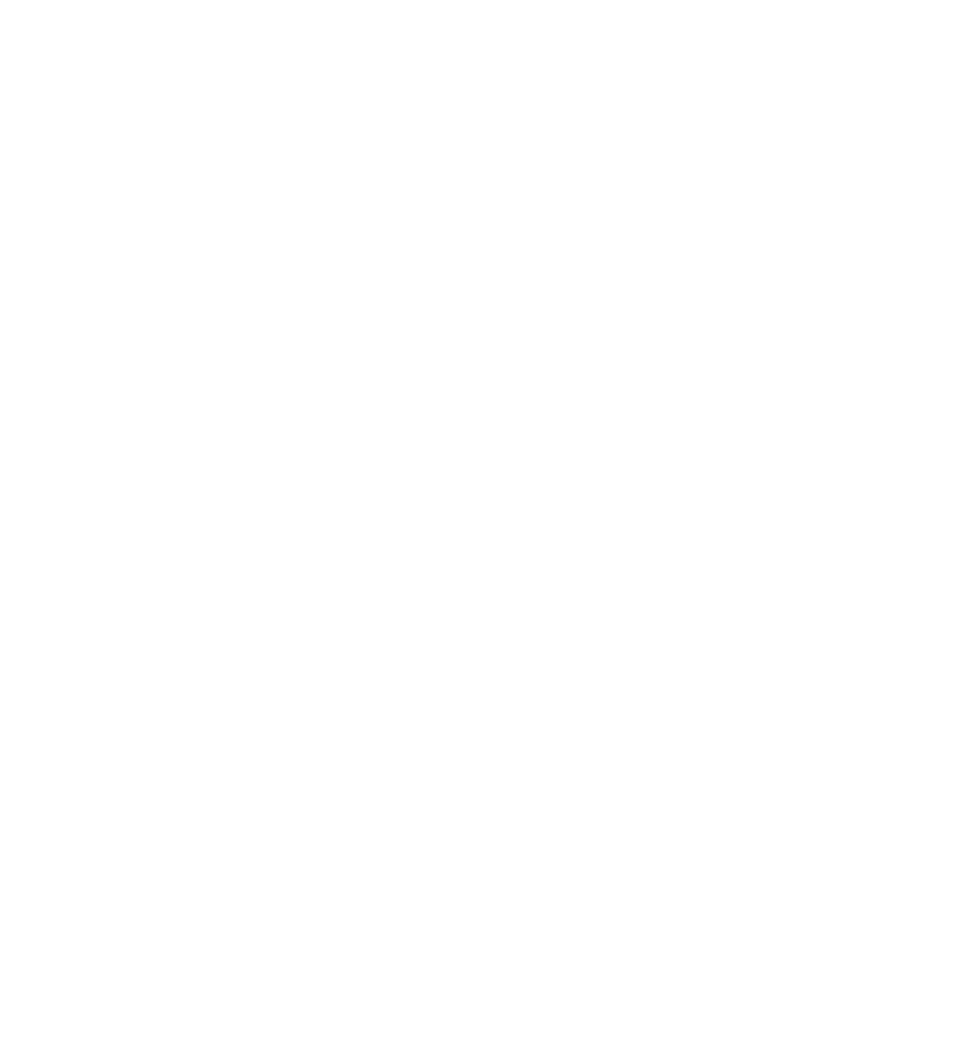 | 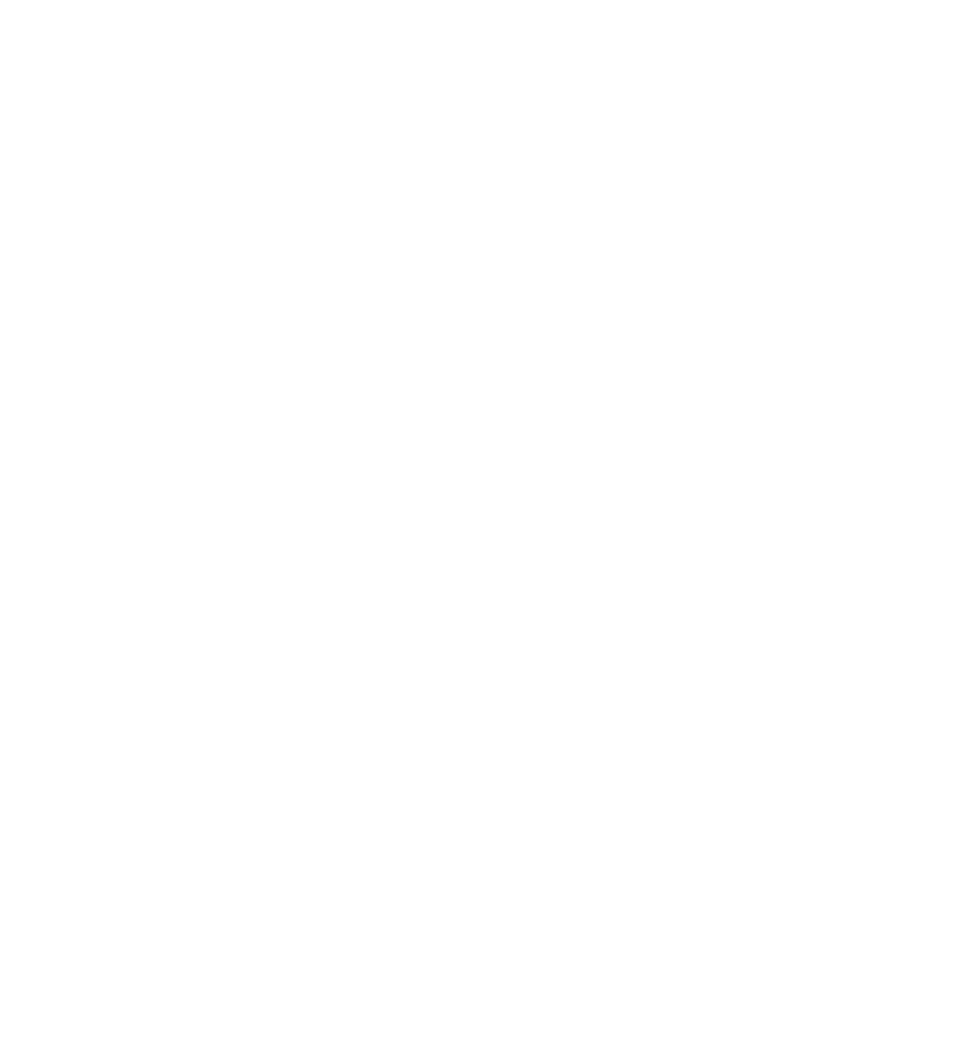 | 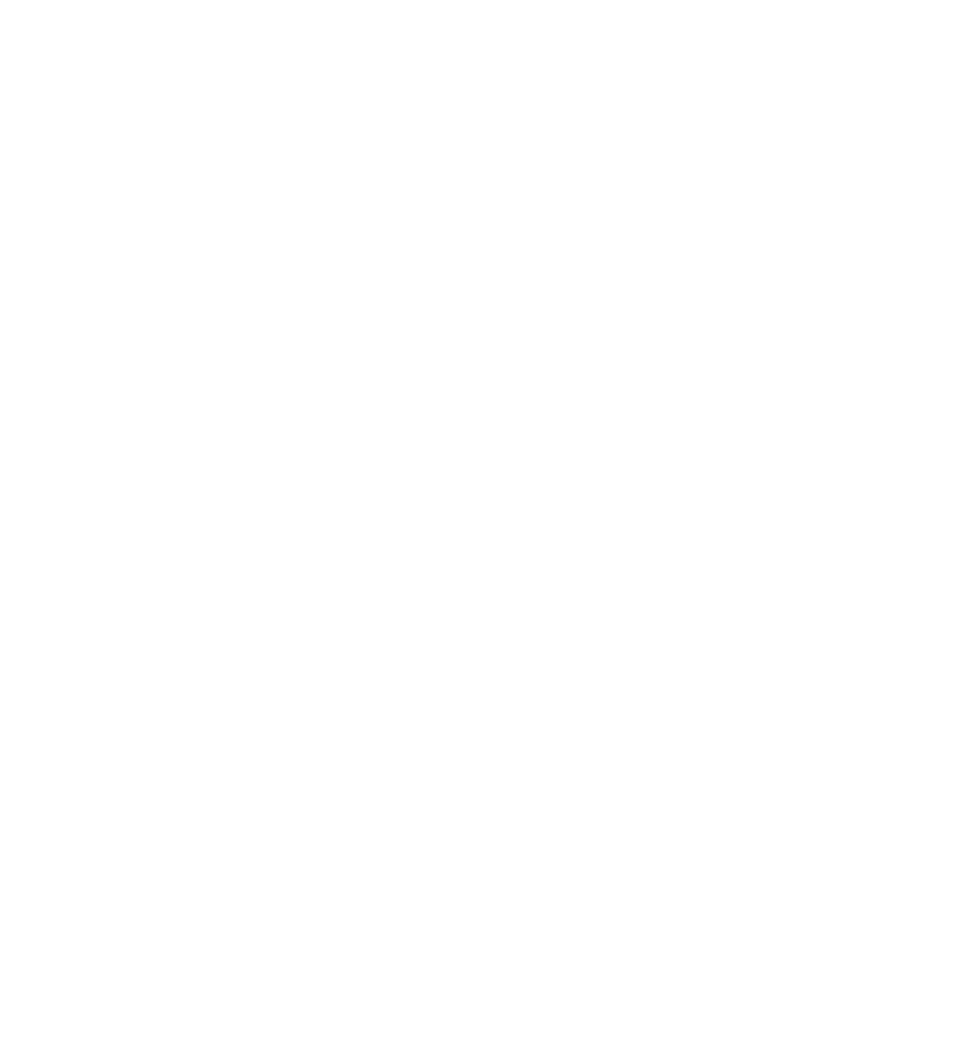 | 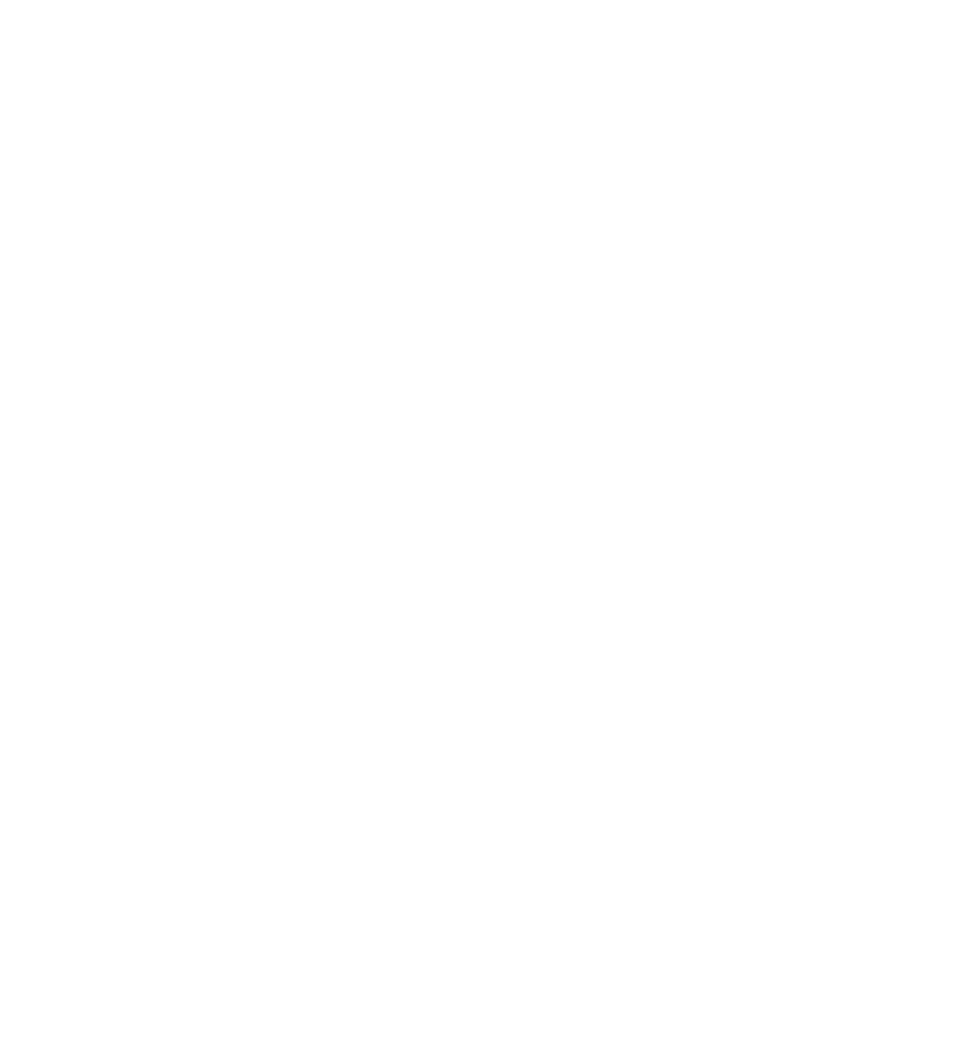 | 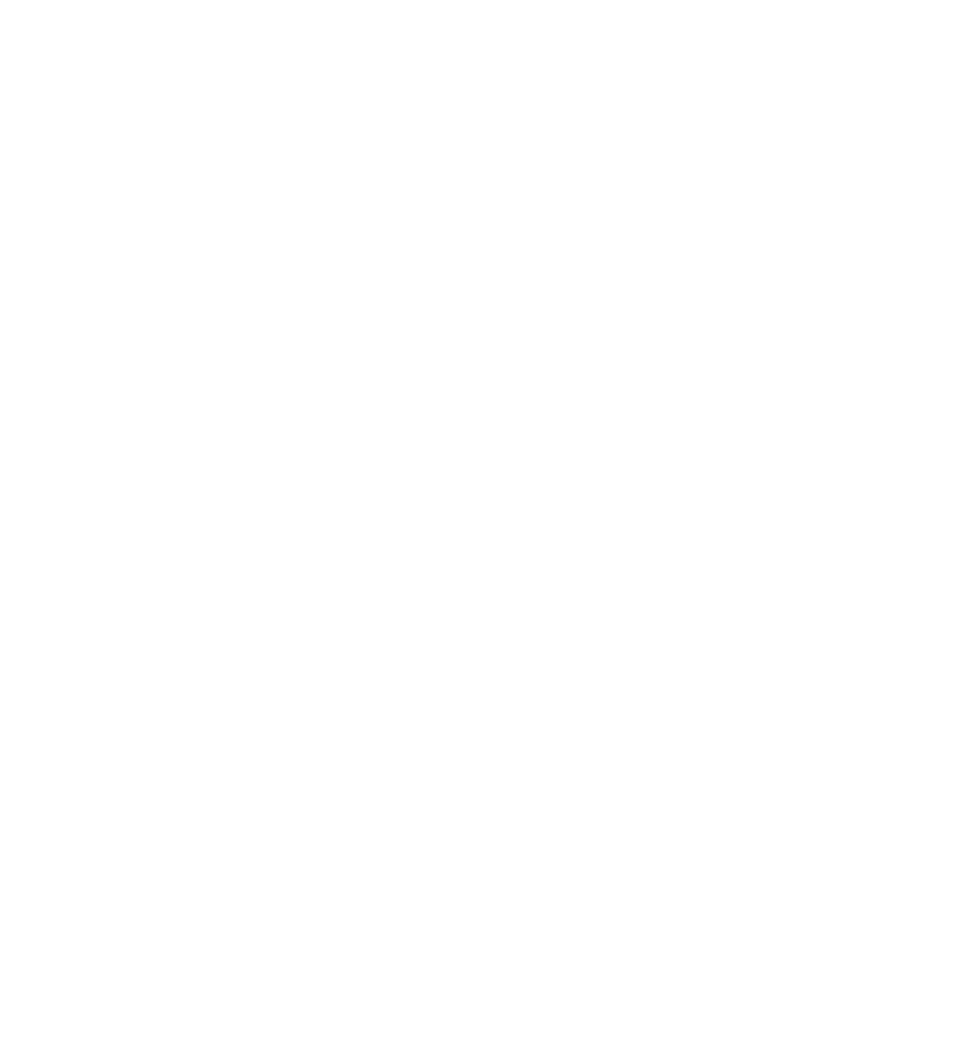 | Extremely satisfied |

**How satisfied are you with Treasure Hunters’ ease of use?**Think about how easy it was for you to use the game for training your speech.

|  | 1 | 2 | 3 | 4 | 5 | 6 | 7 | 8 | 9 | 10 |  |
| --- | --- | --- | --- | --- | --- | --- | --- | --- | --- | --- | --- |
| Extremely unsatisfied | 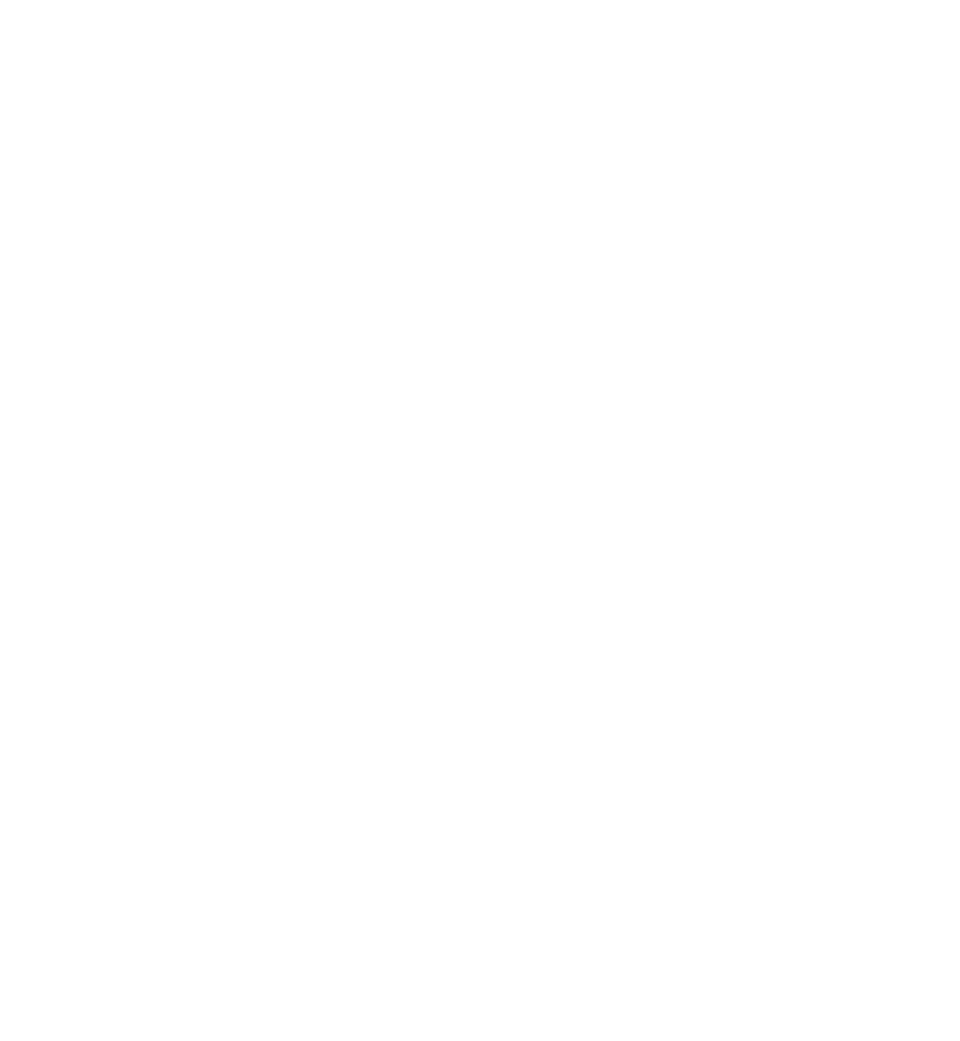 | 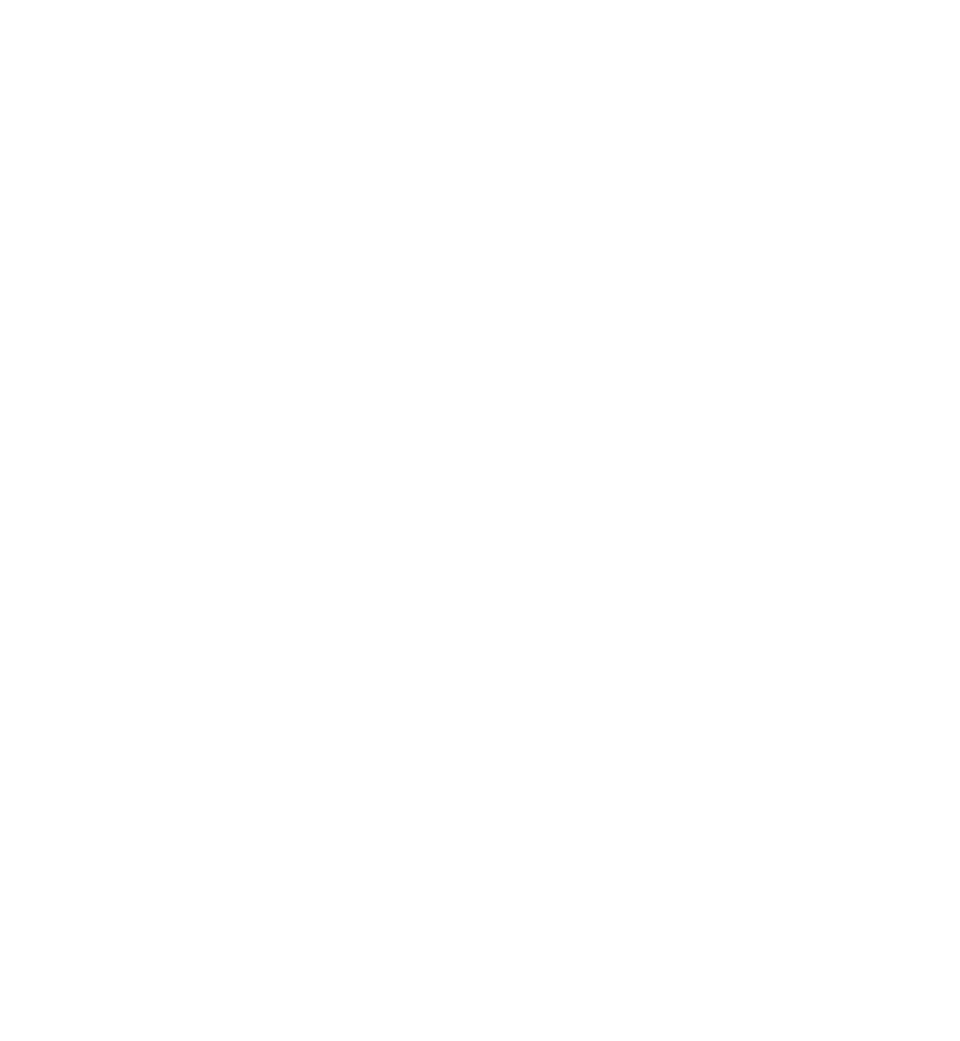 | 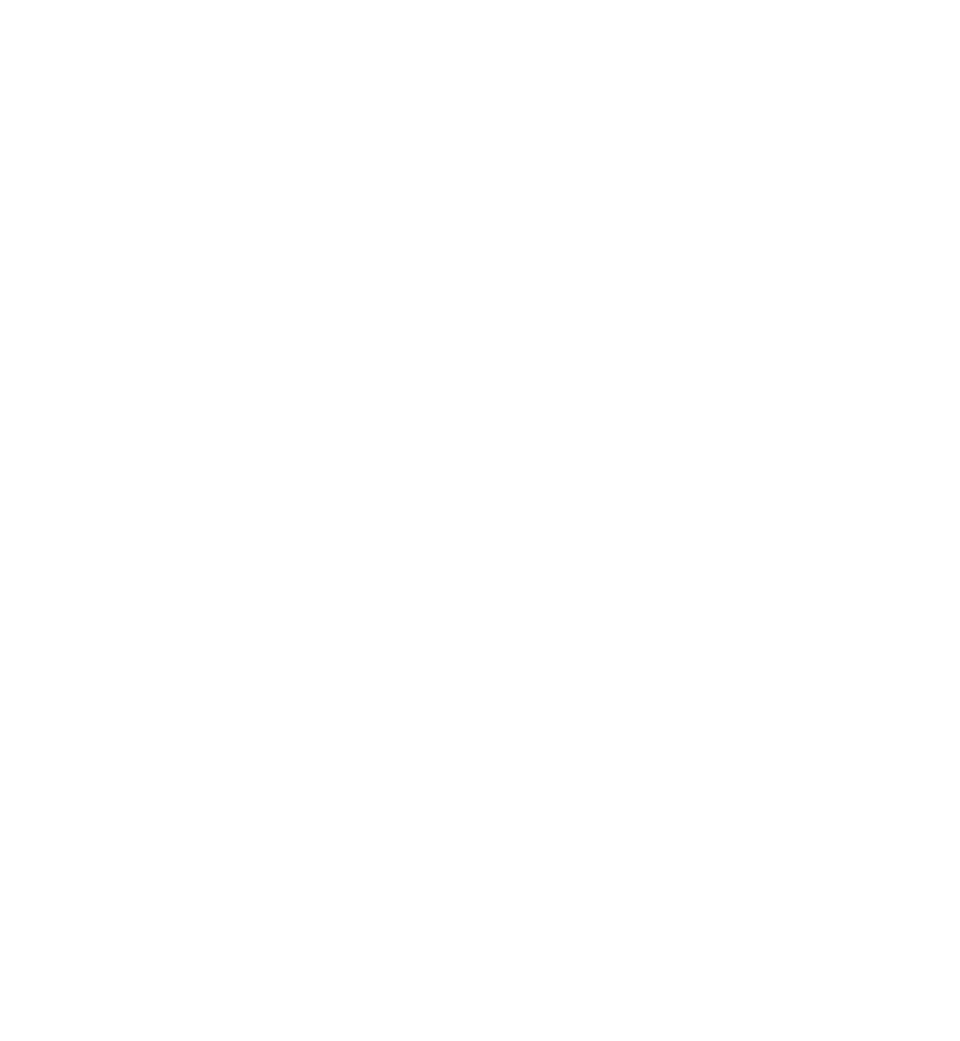 | 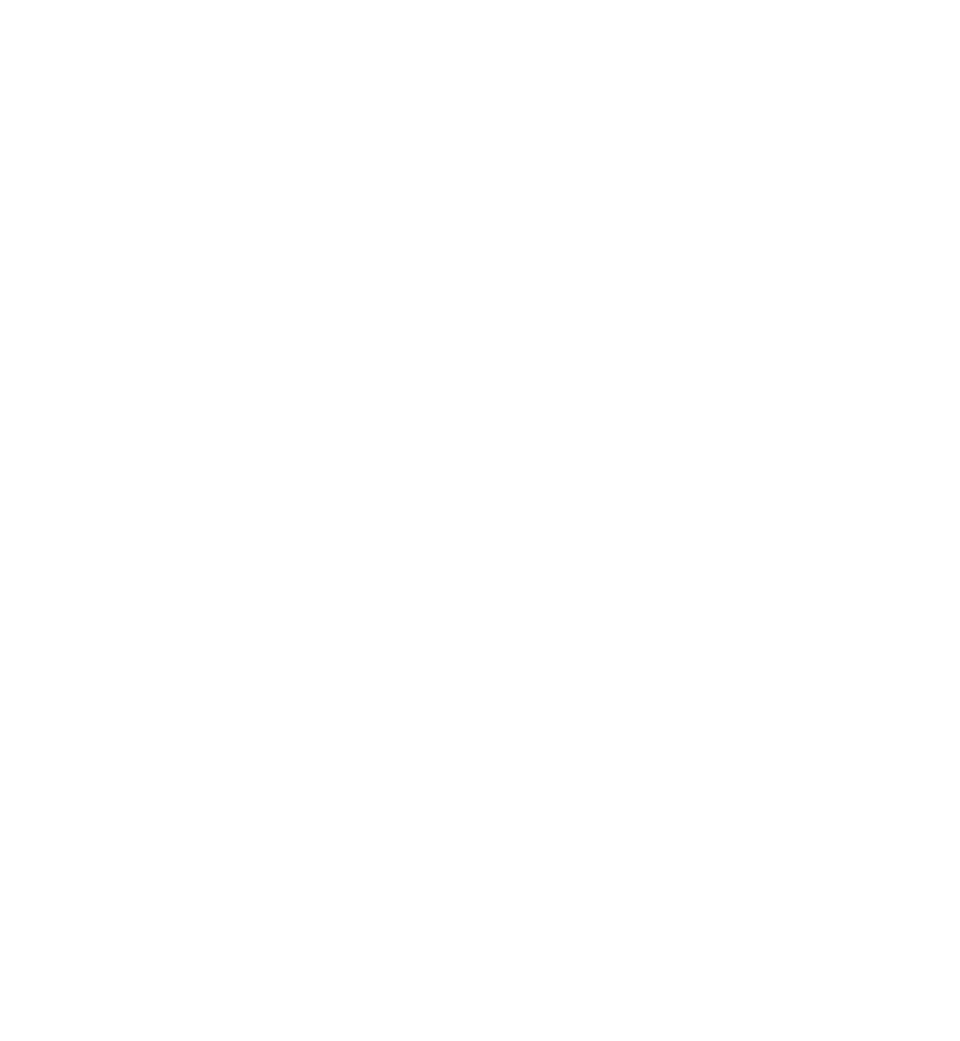 | 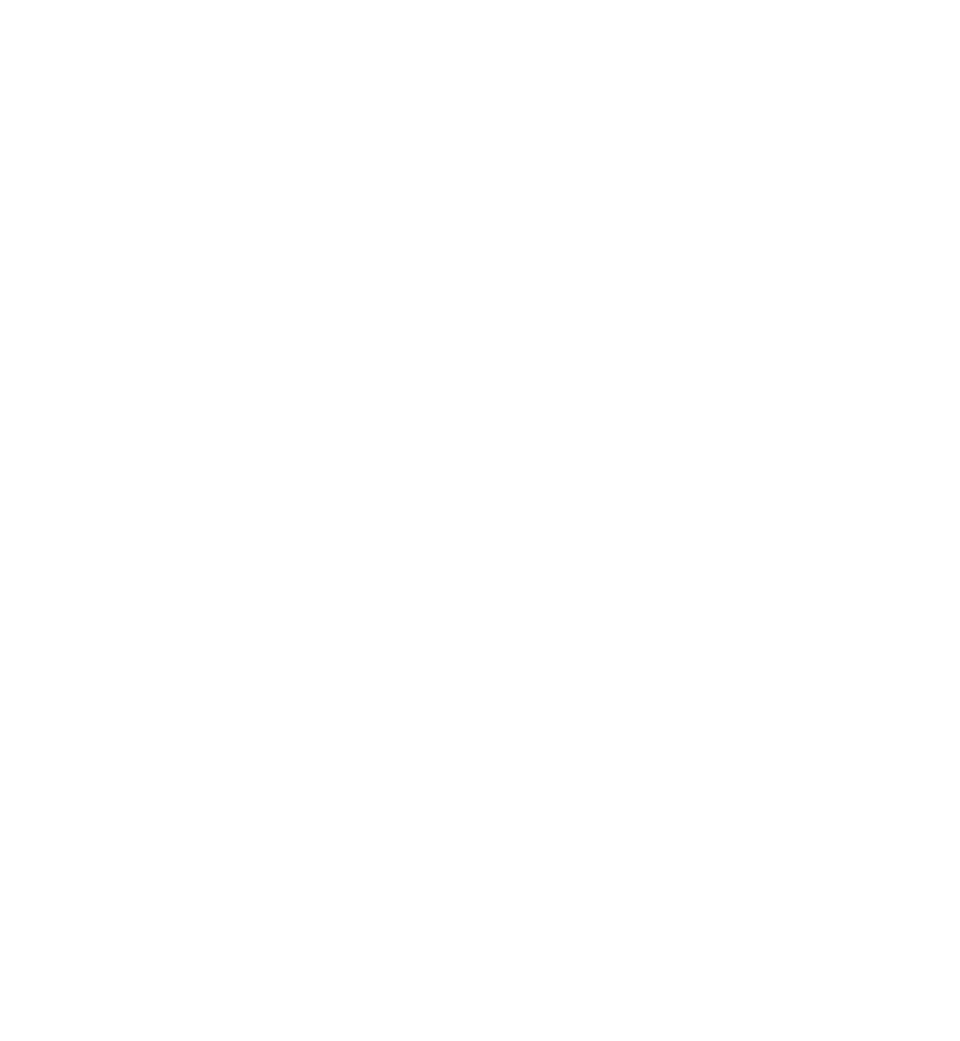 | 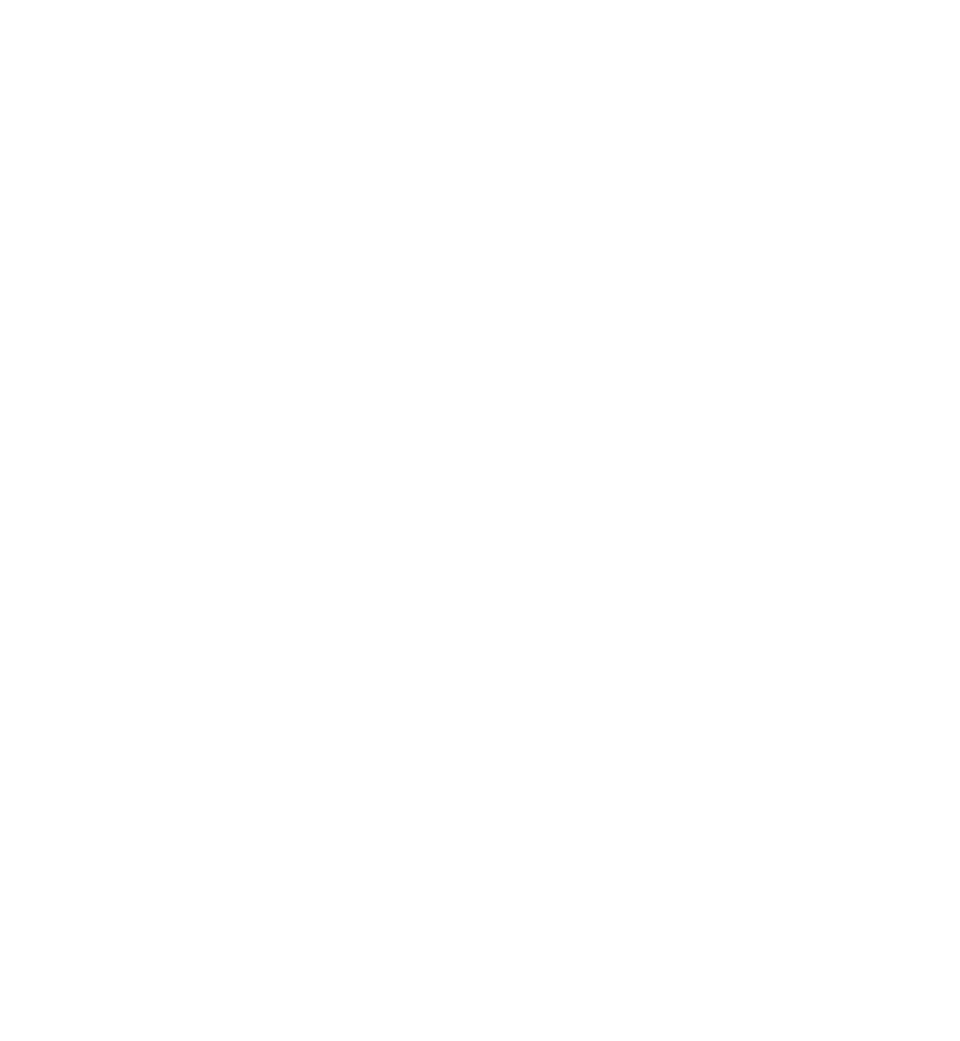 | 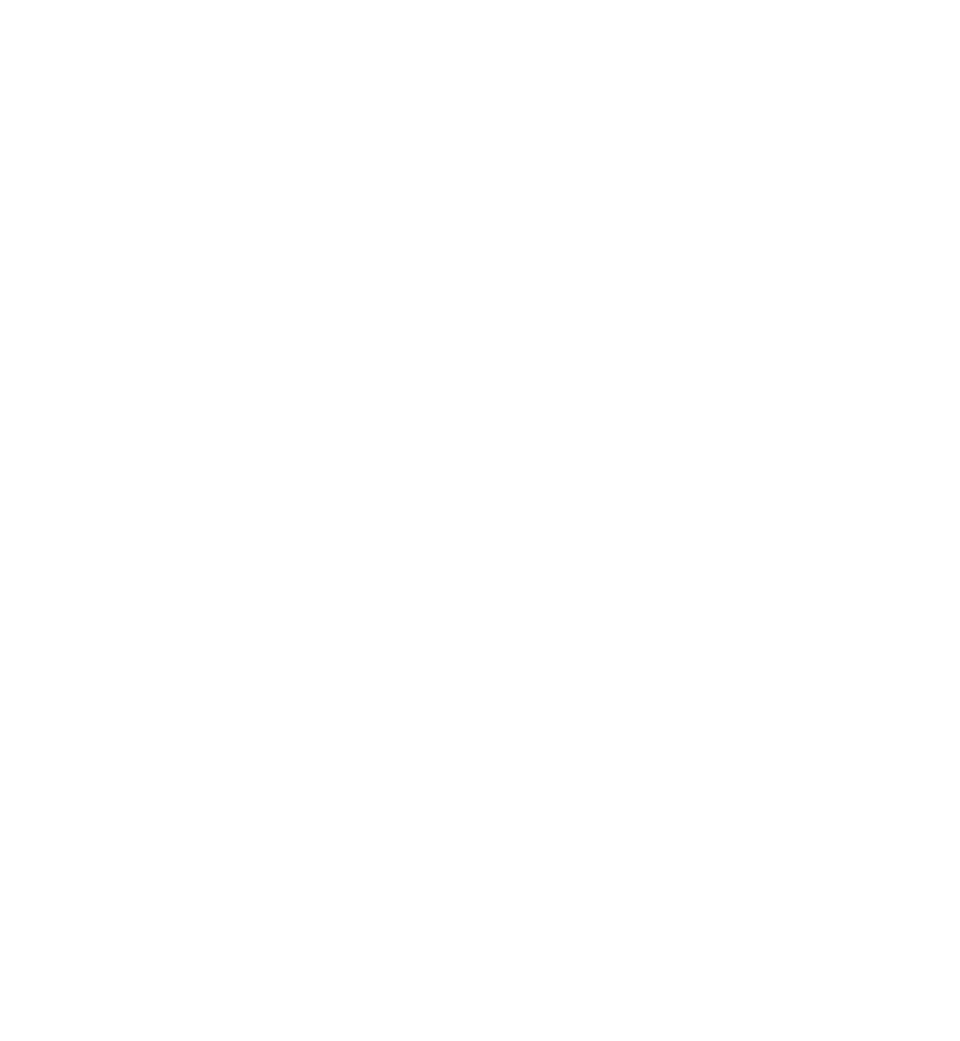 | 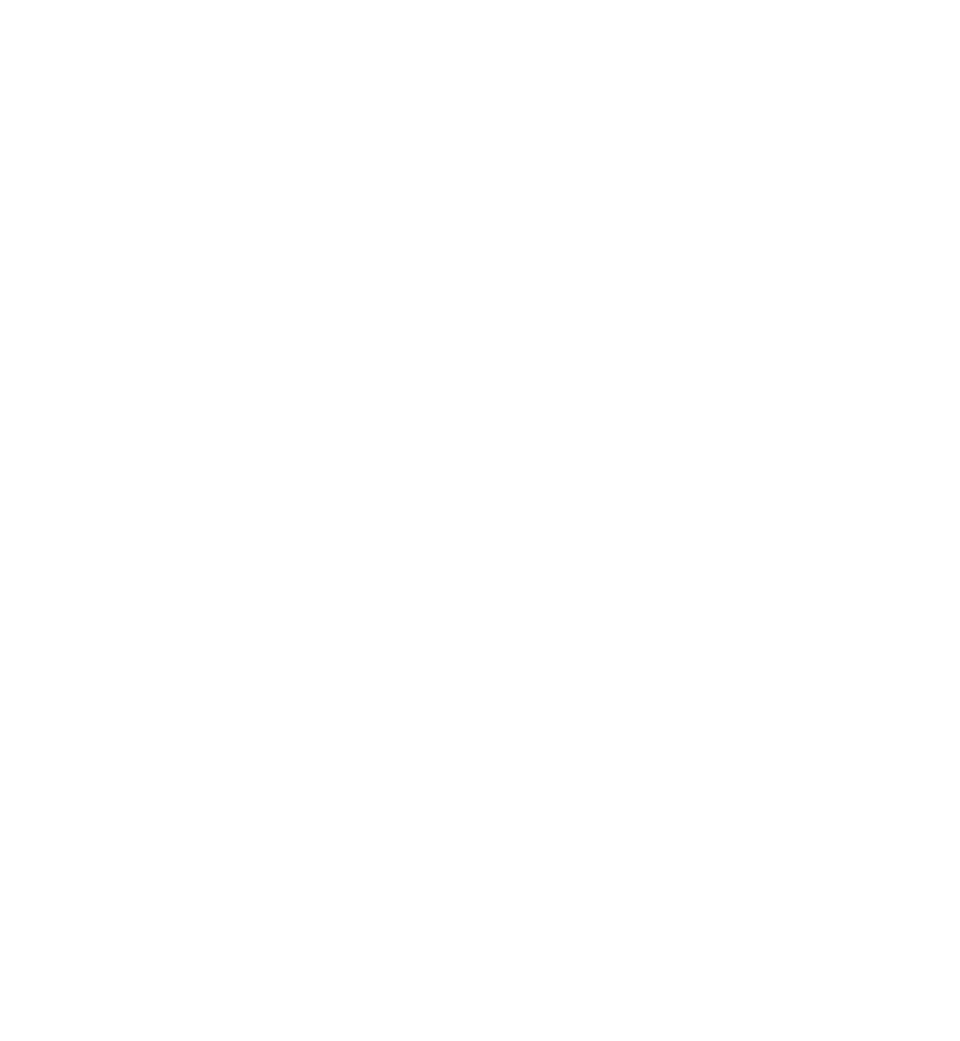 | 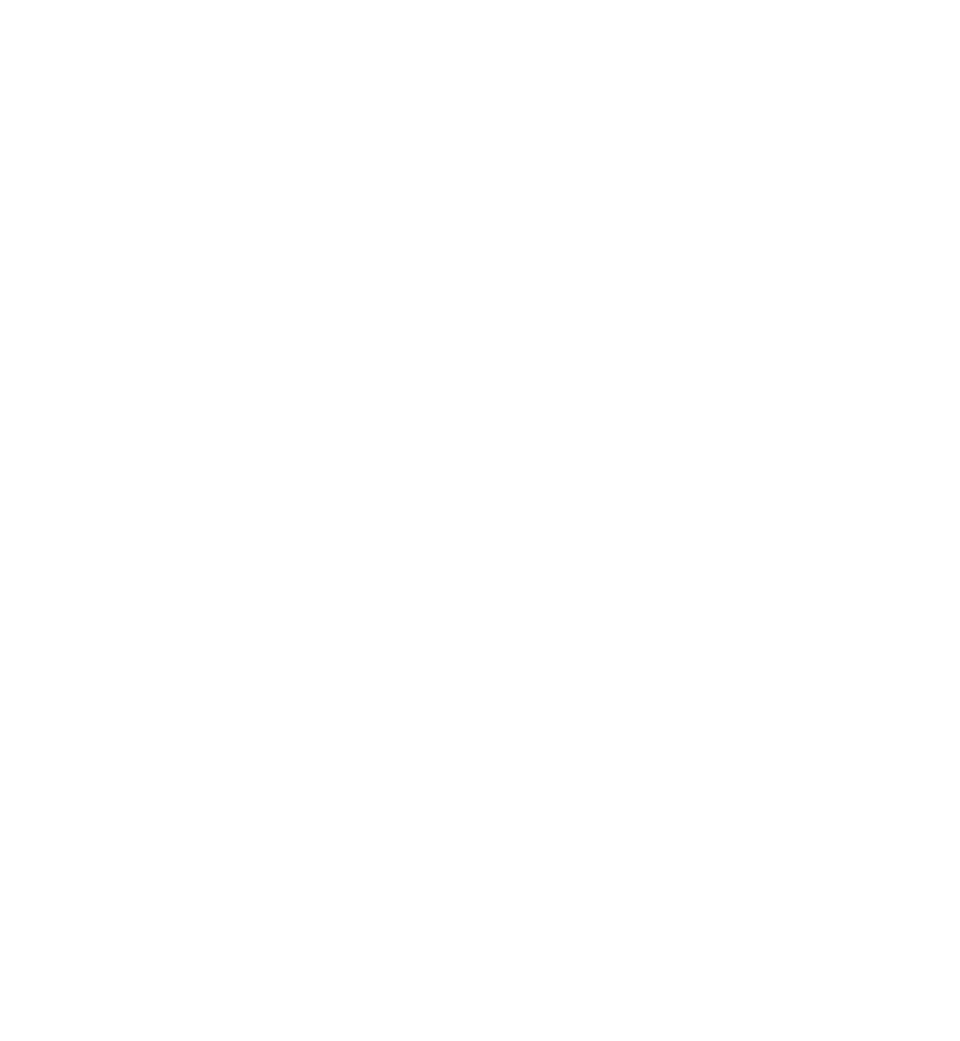 | 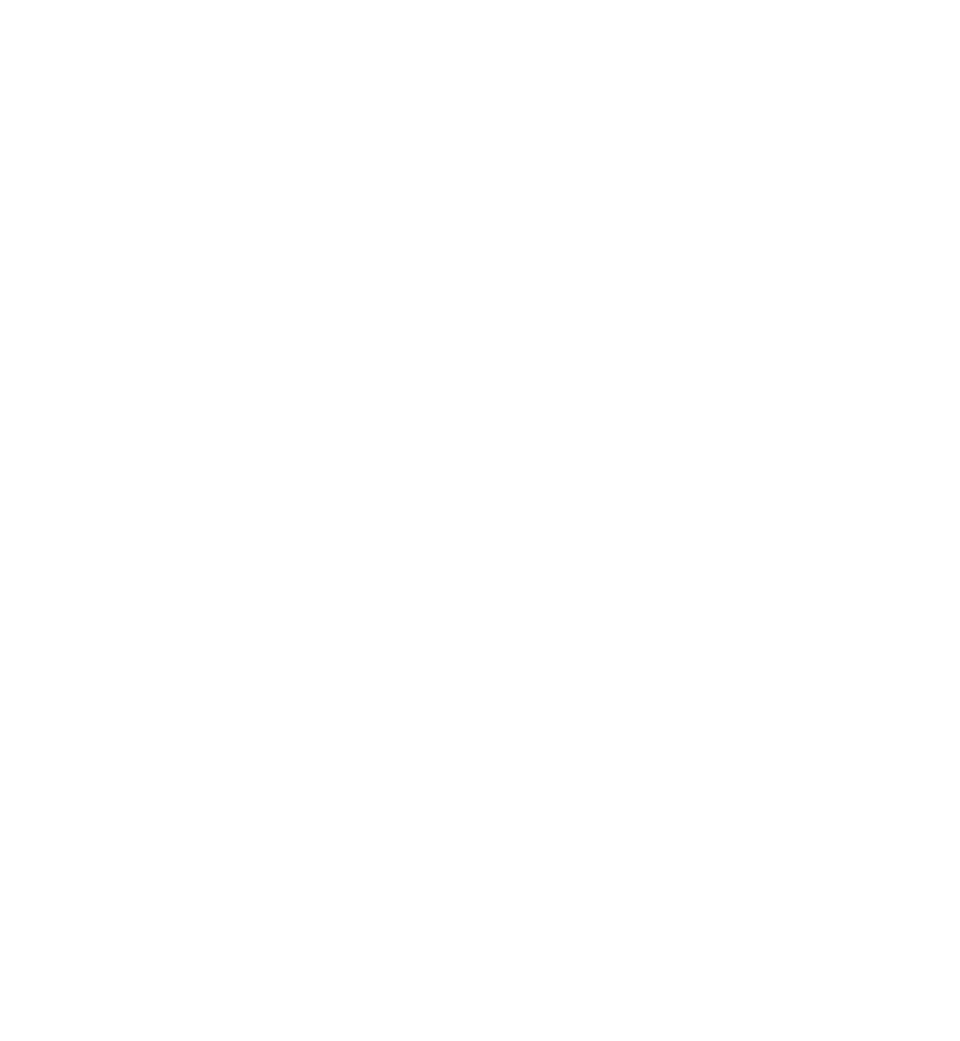 | Extremely satisfied |

**How satisfied are you about Treasure Hunters’ attractiveness?**Think about how the game’s visuals appealed to you.

|  | 1 | 2 | 3 | 4 | 5 | 6 | 7 | 8 | 9 | 10 |  |
| --- | --- | --- | --- | --- | --- | --- | --- | --- | --- | --- | --- |
| Extremely unsatisfied | 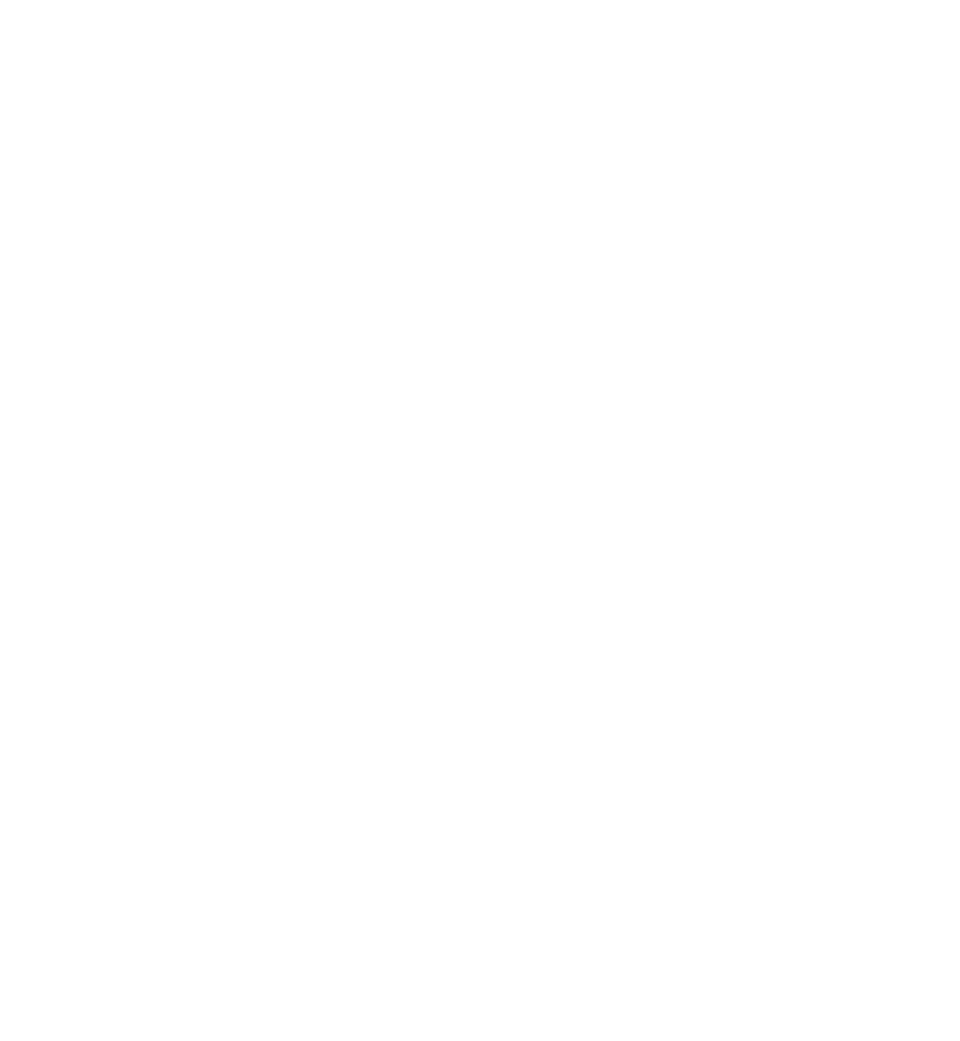 | 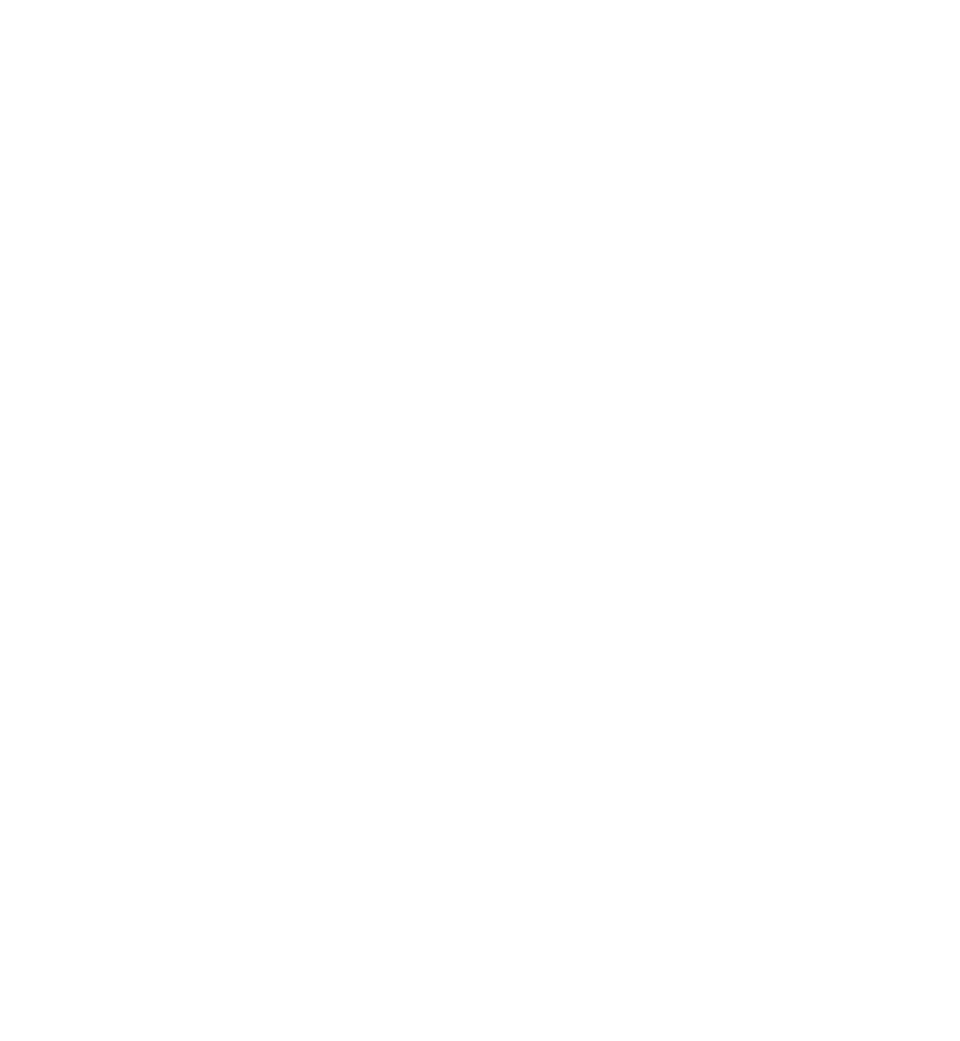 | 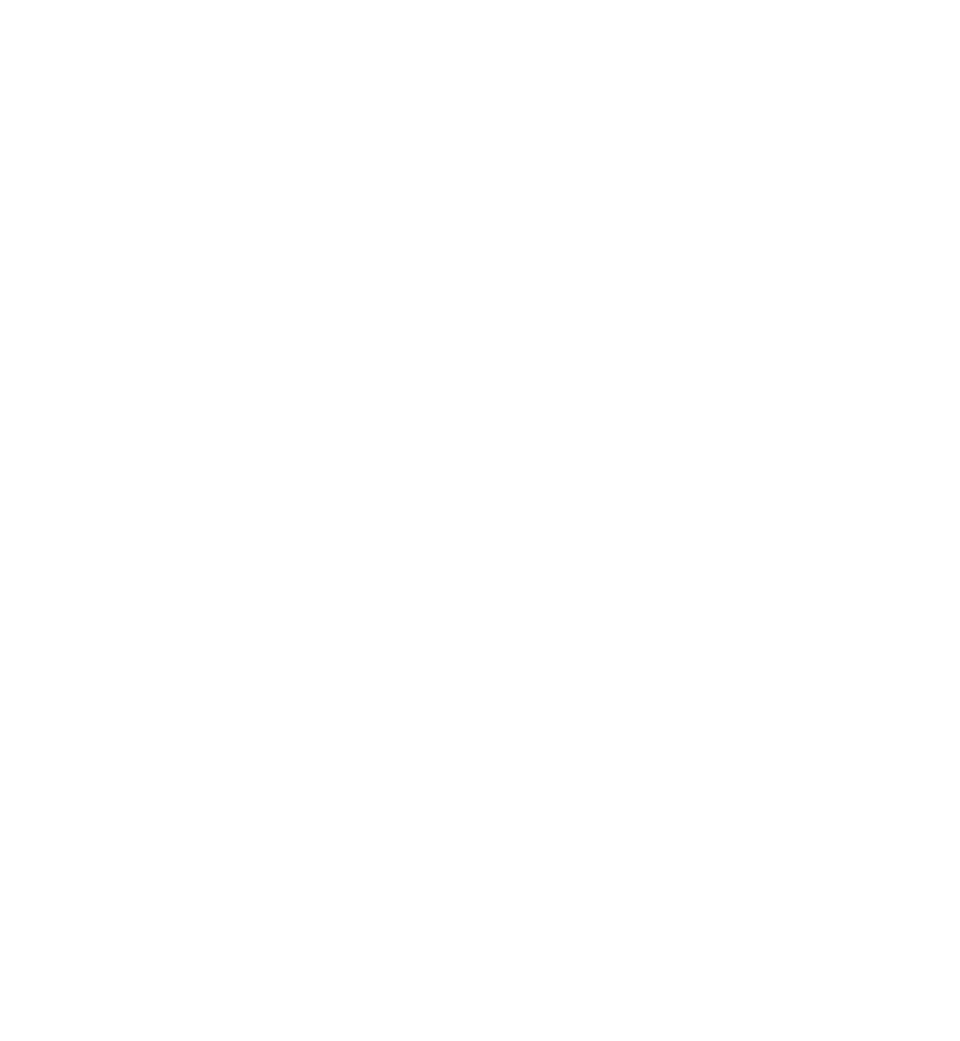 | 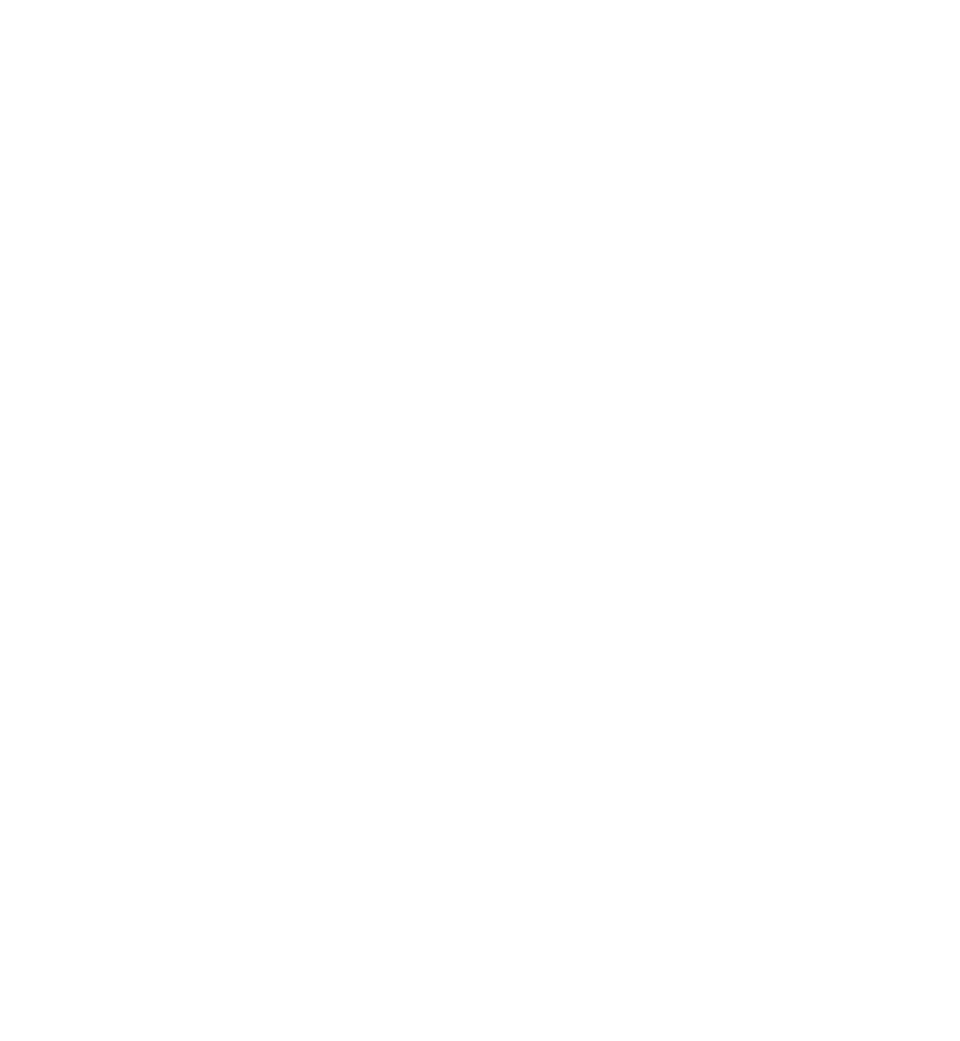 | 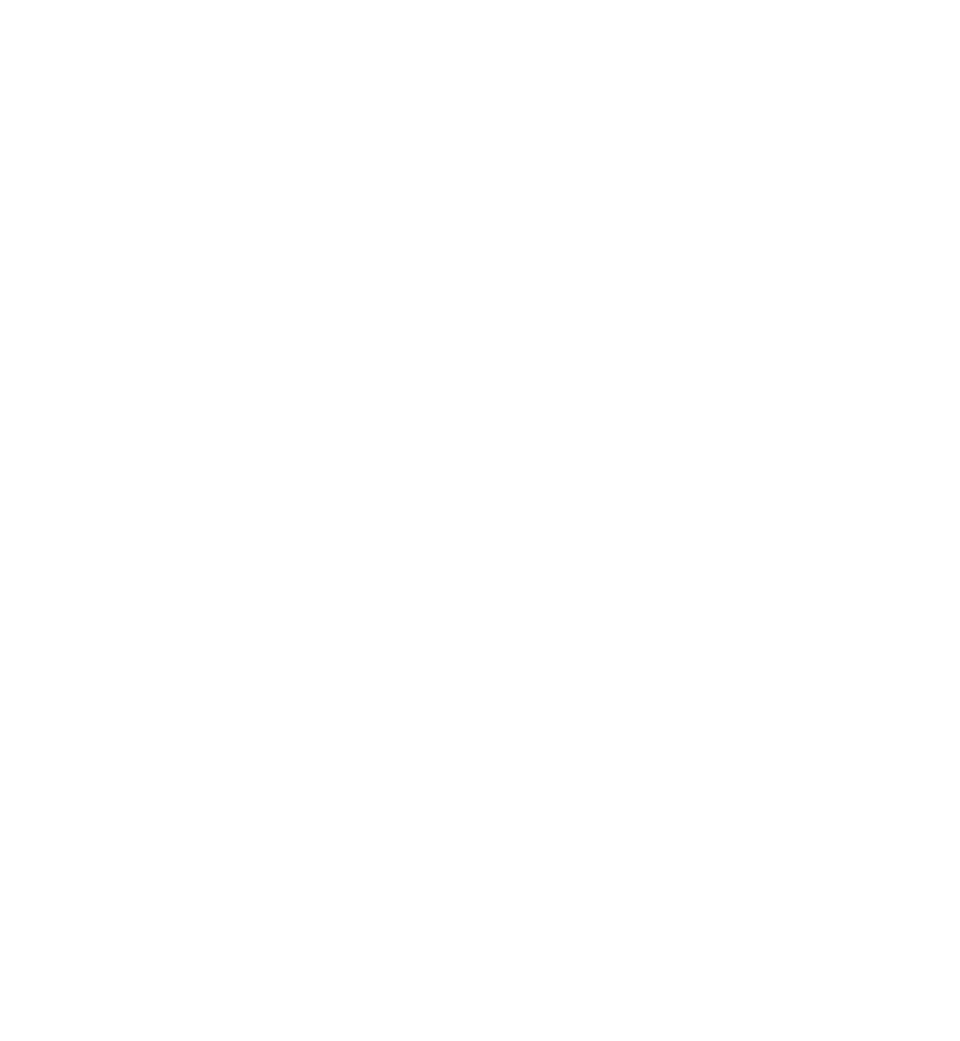 | 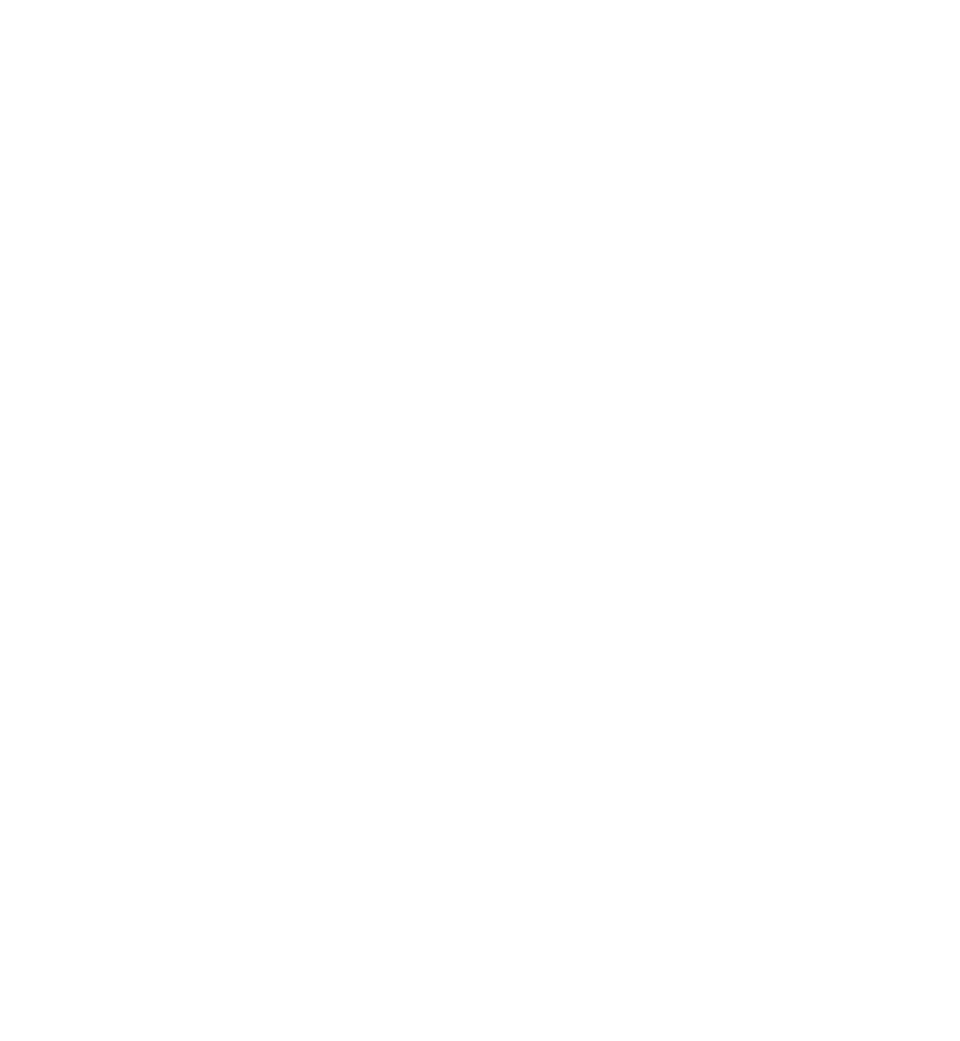 | 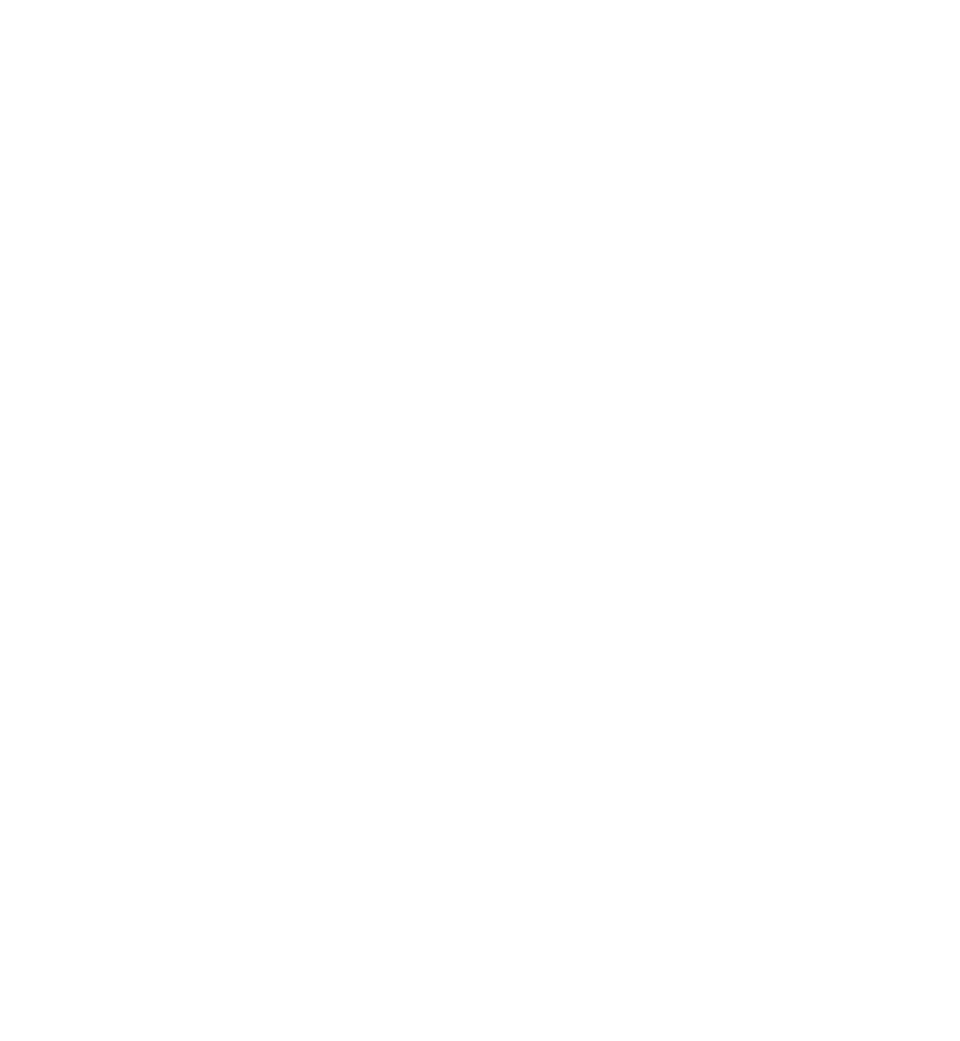 | 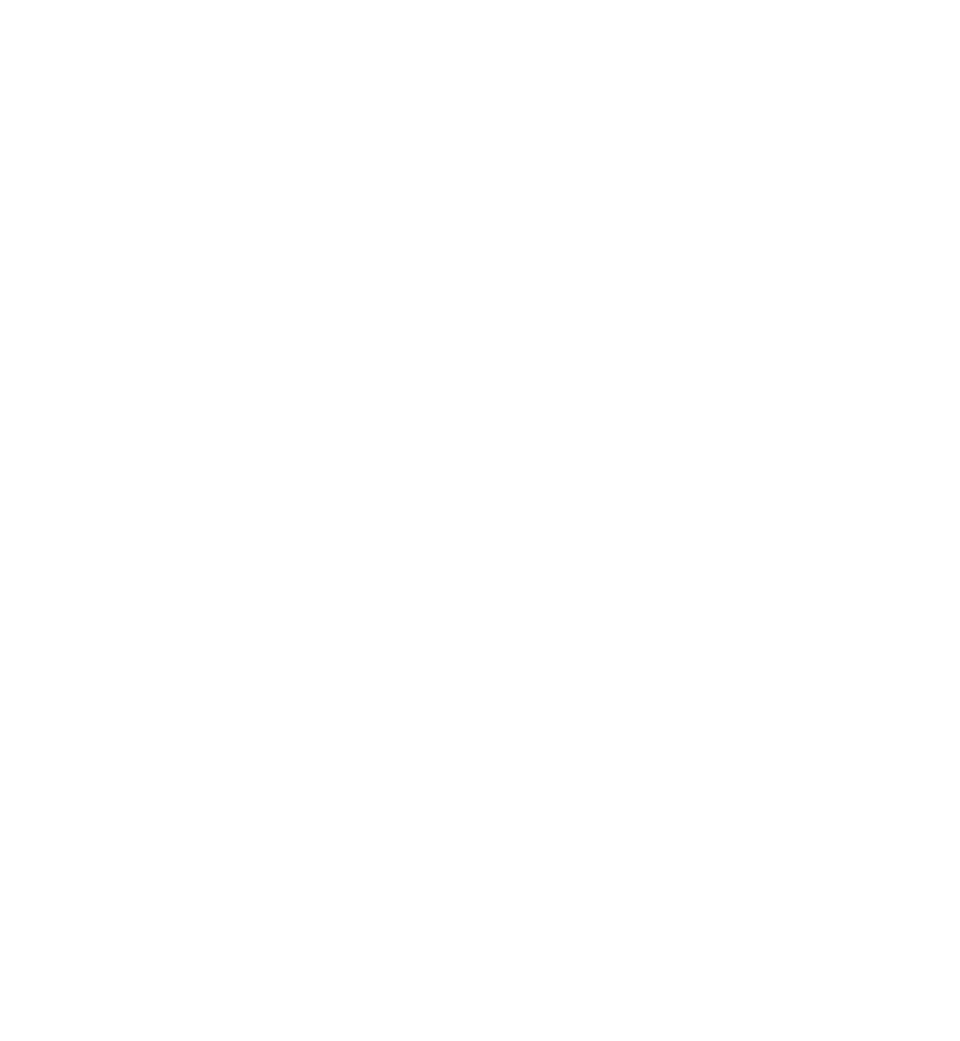 | 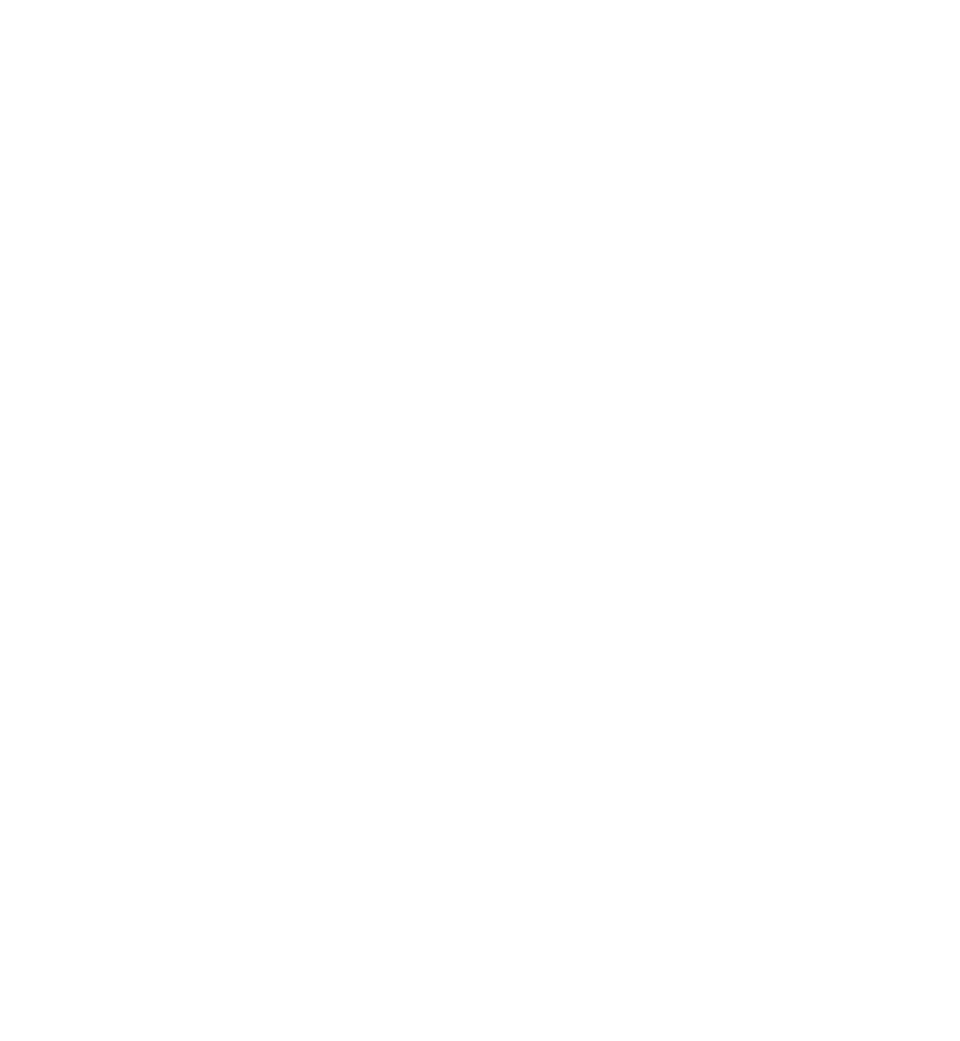 | 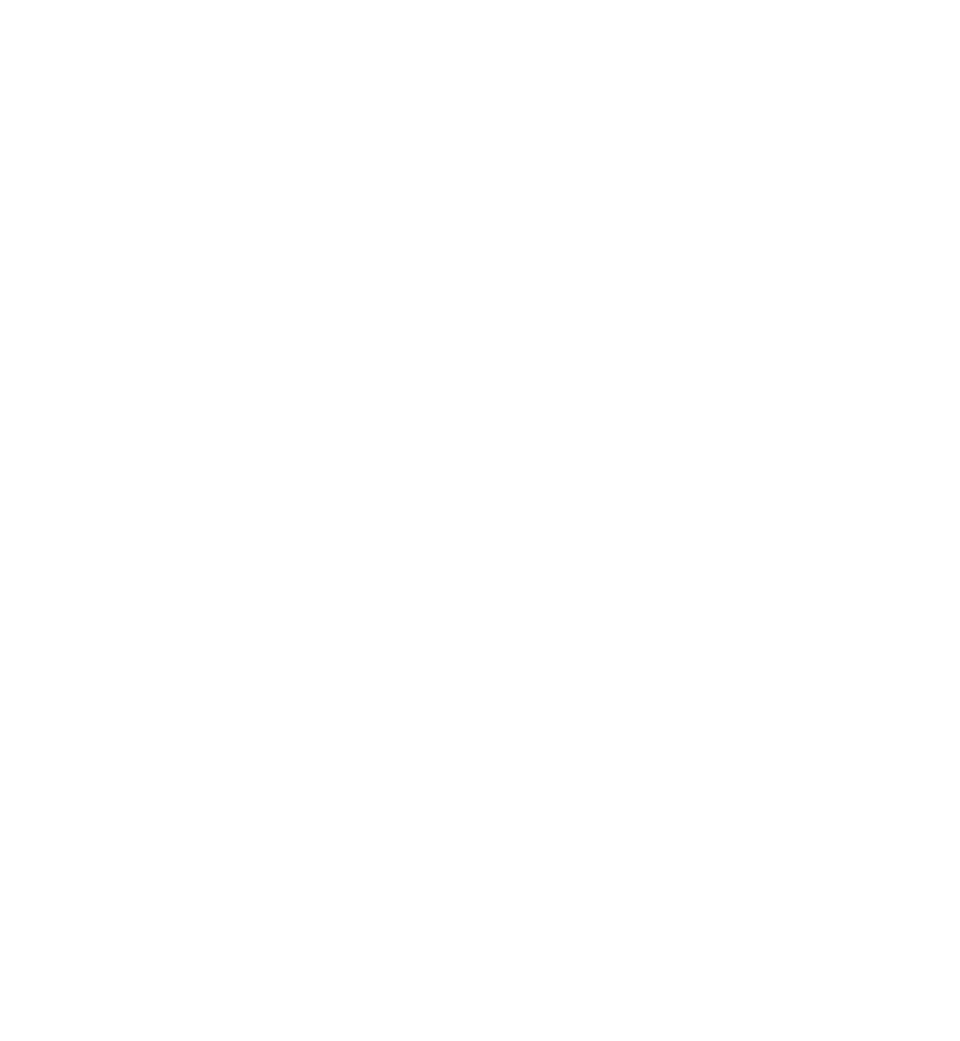 | Extremely satisfied |

**How satisfied are you in general about Treasure Hunters as a game for speech training?**

|  | 1 | 2 | 3 | 4 | 5 | 6 | 7 | 8 | 9 | 10 |  |
| --- | --- | --- | --- | --- | --- | --- | --- | --- | --- | --- | --- |
| Extremely unsatisfied | 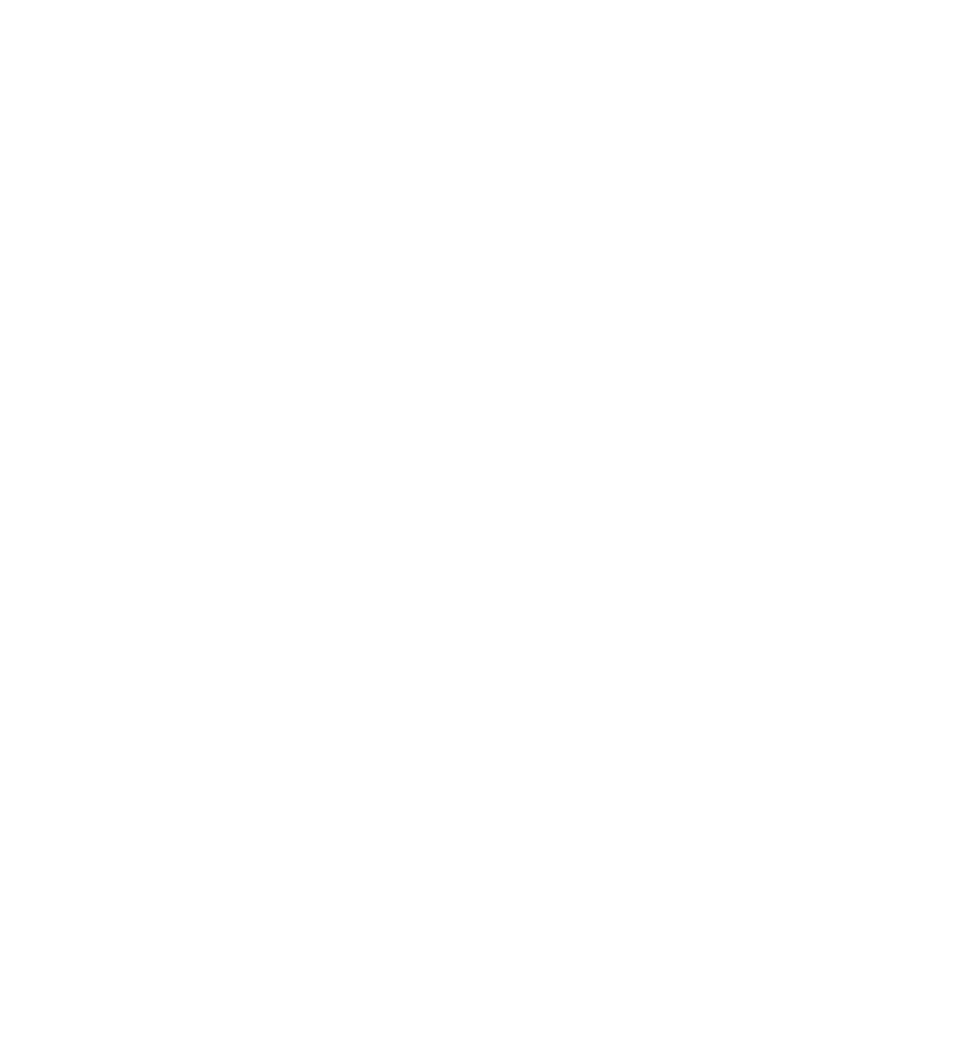 | 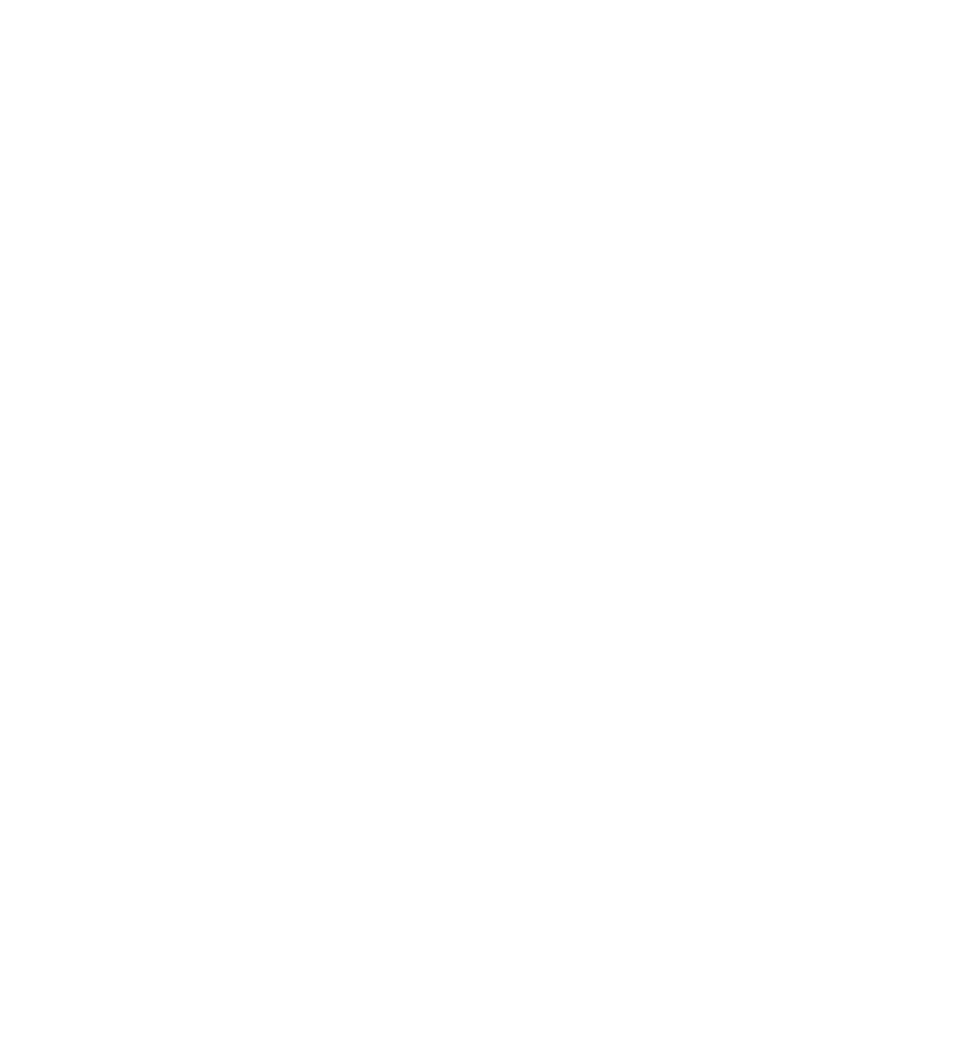 | 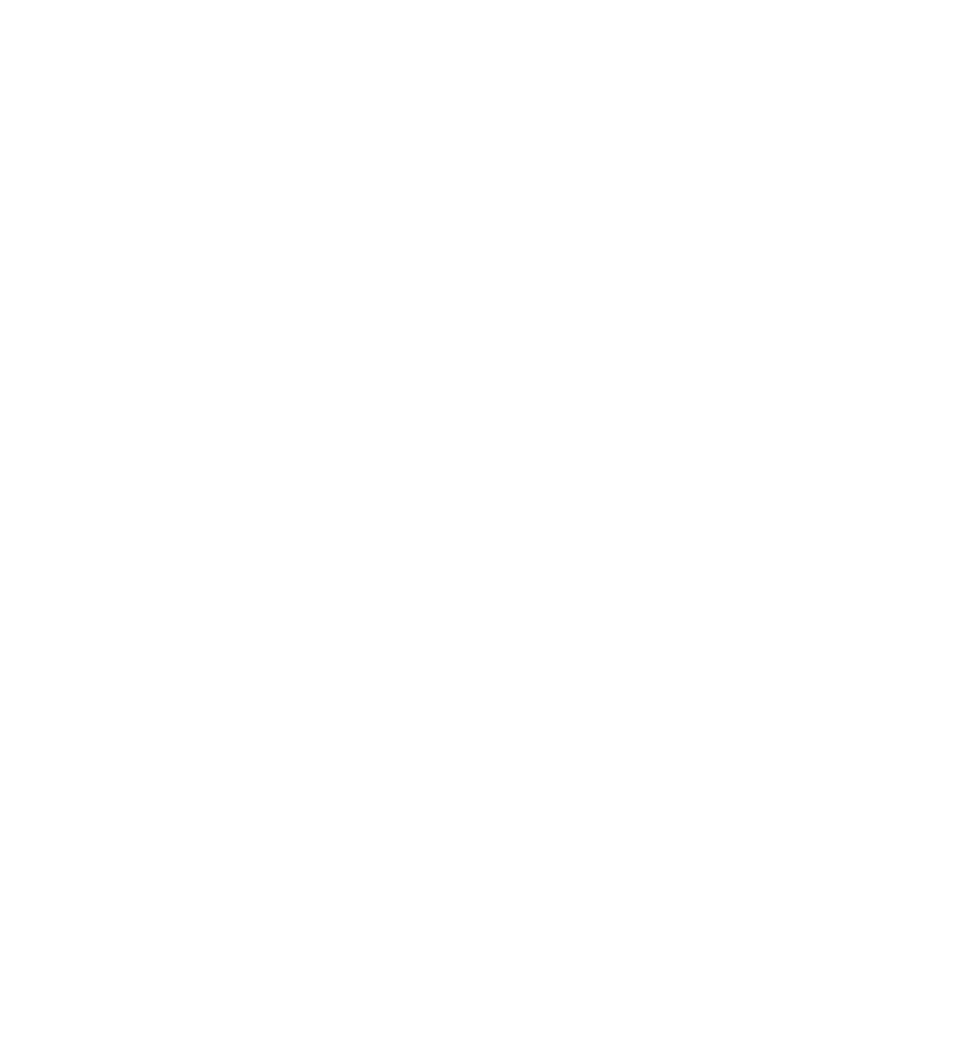 | 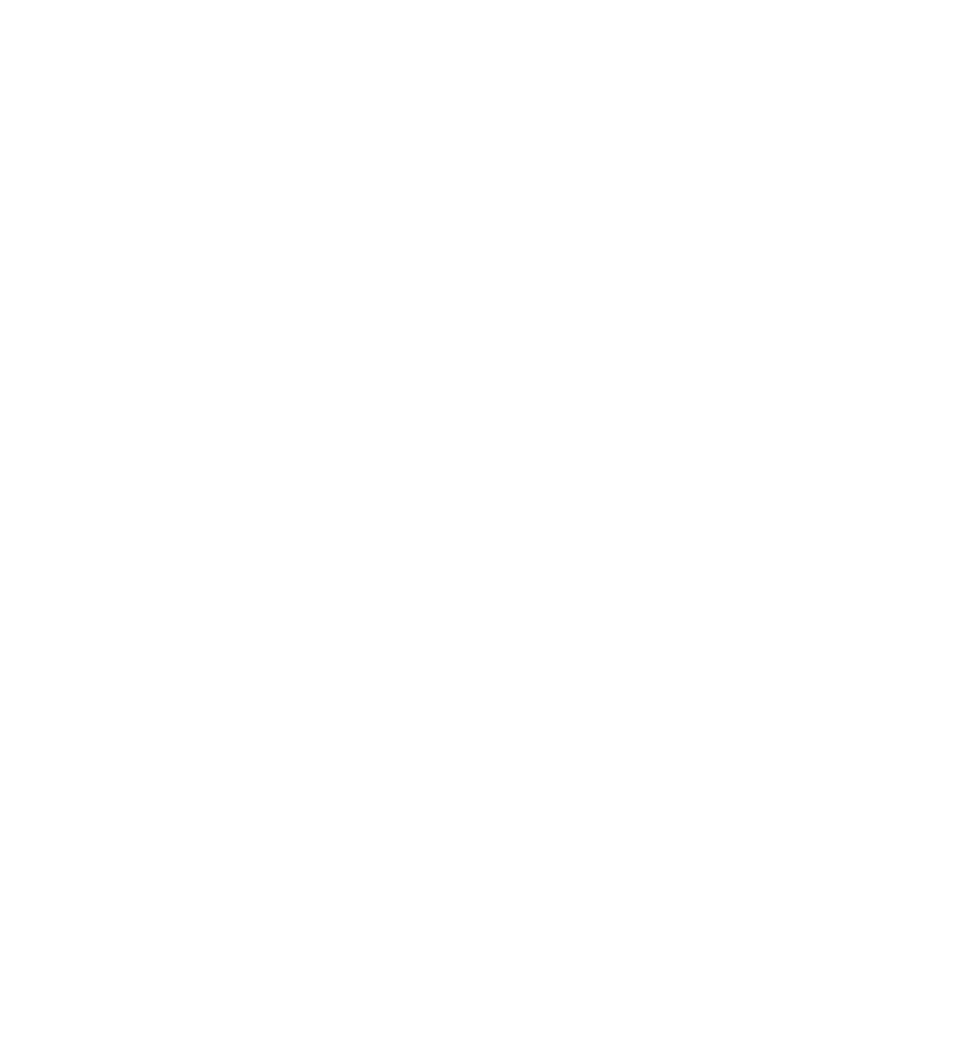 | 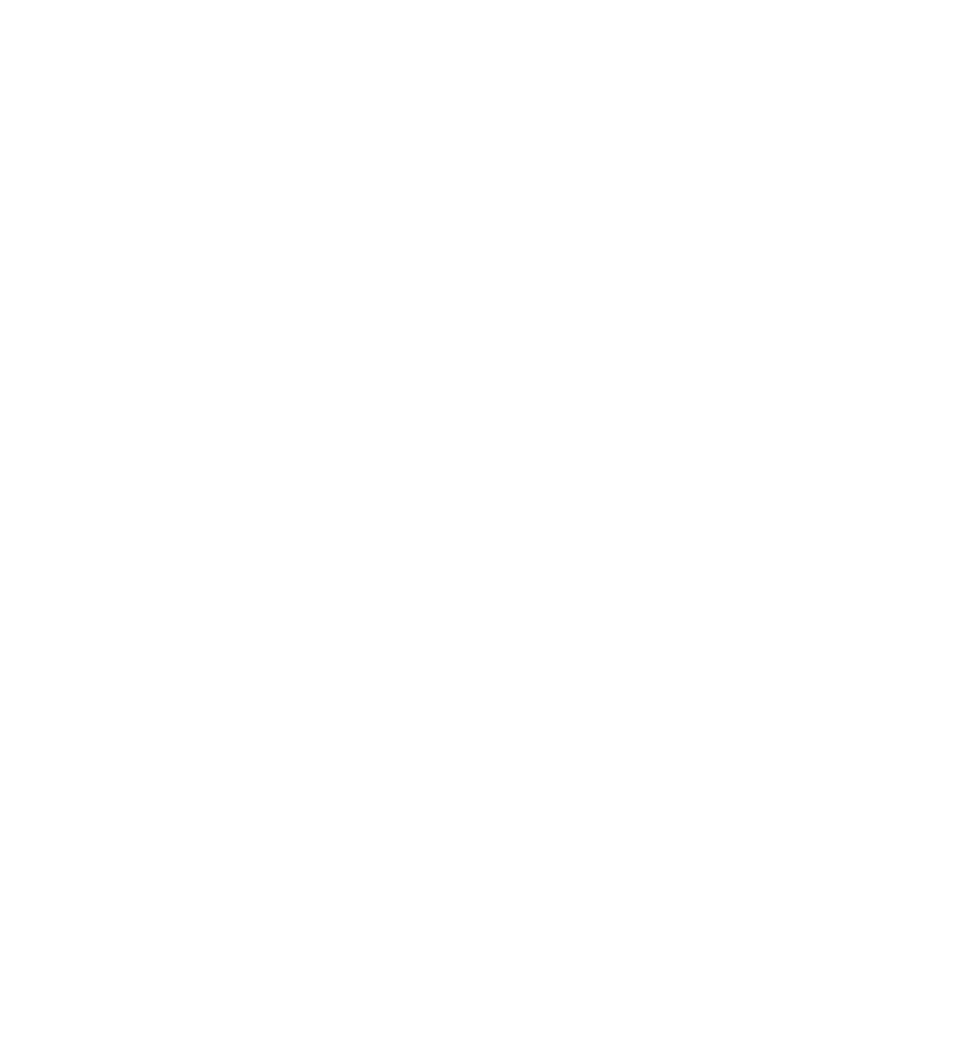 | 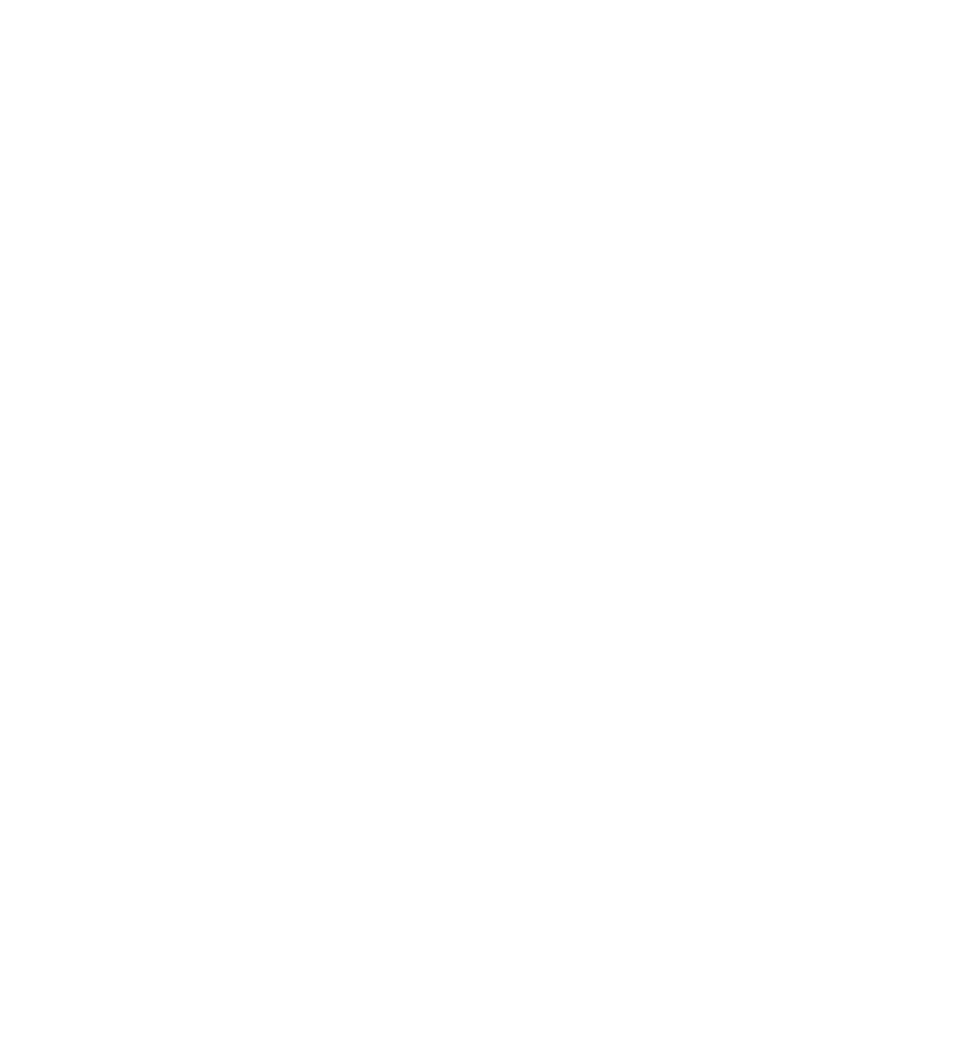 | 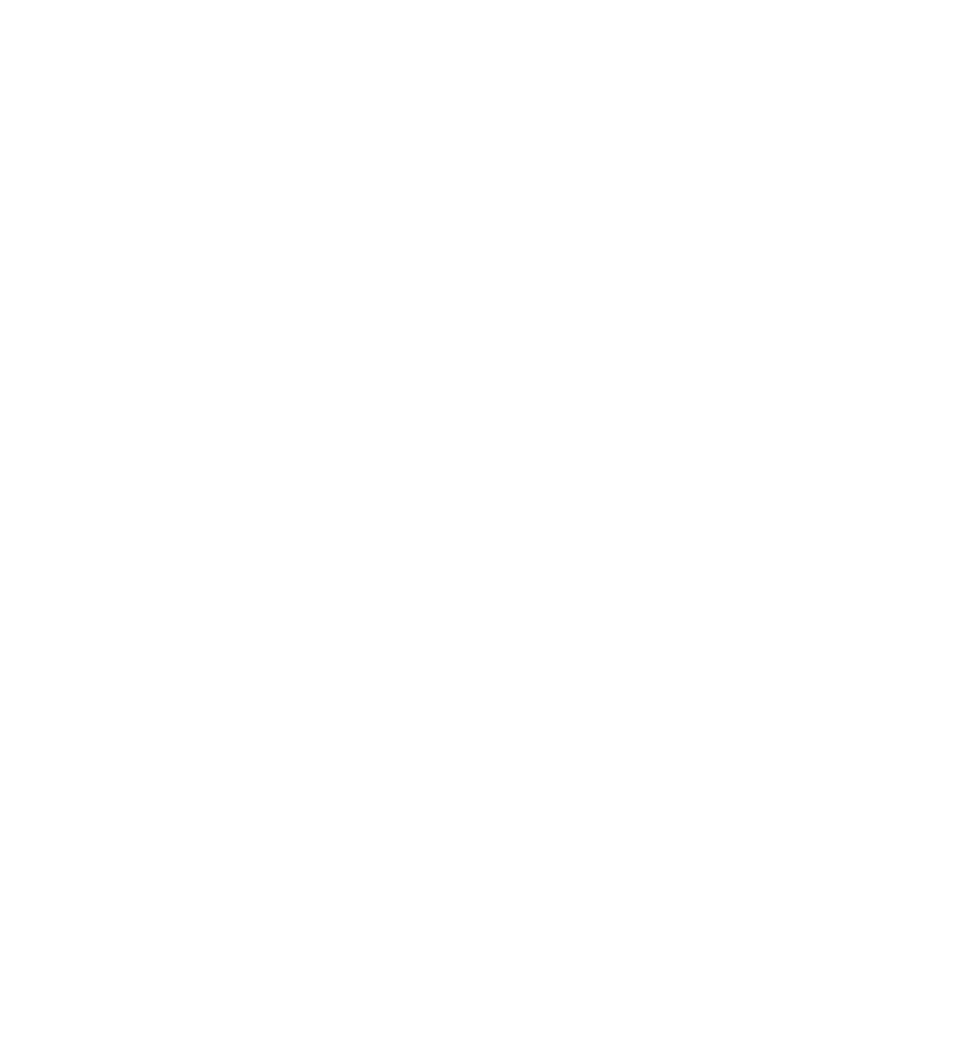 | 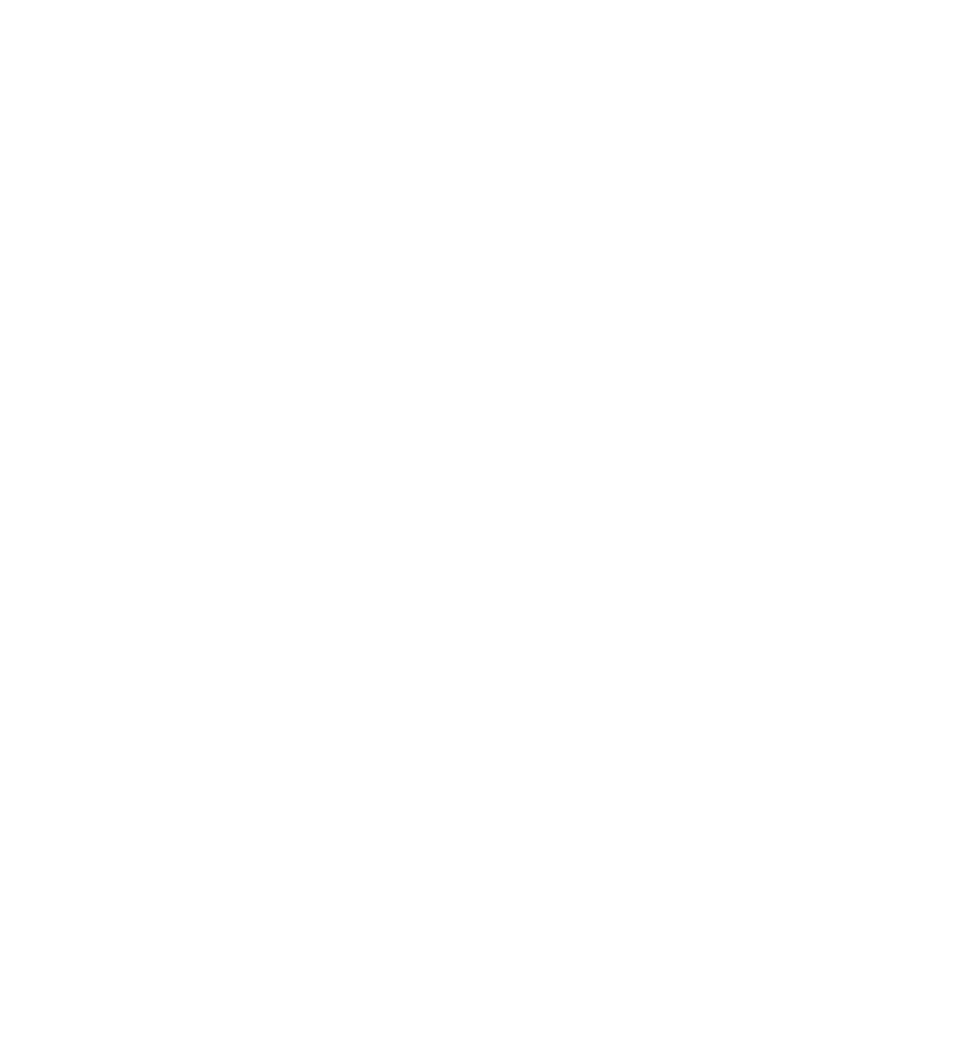 | 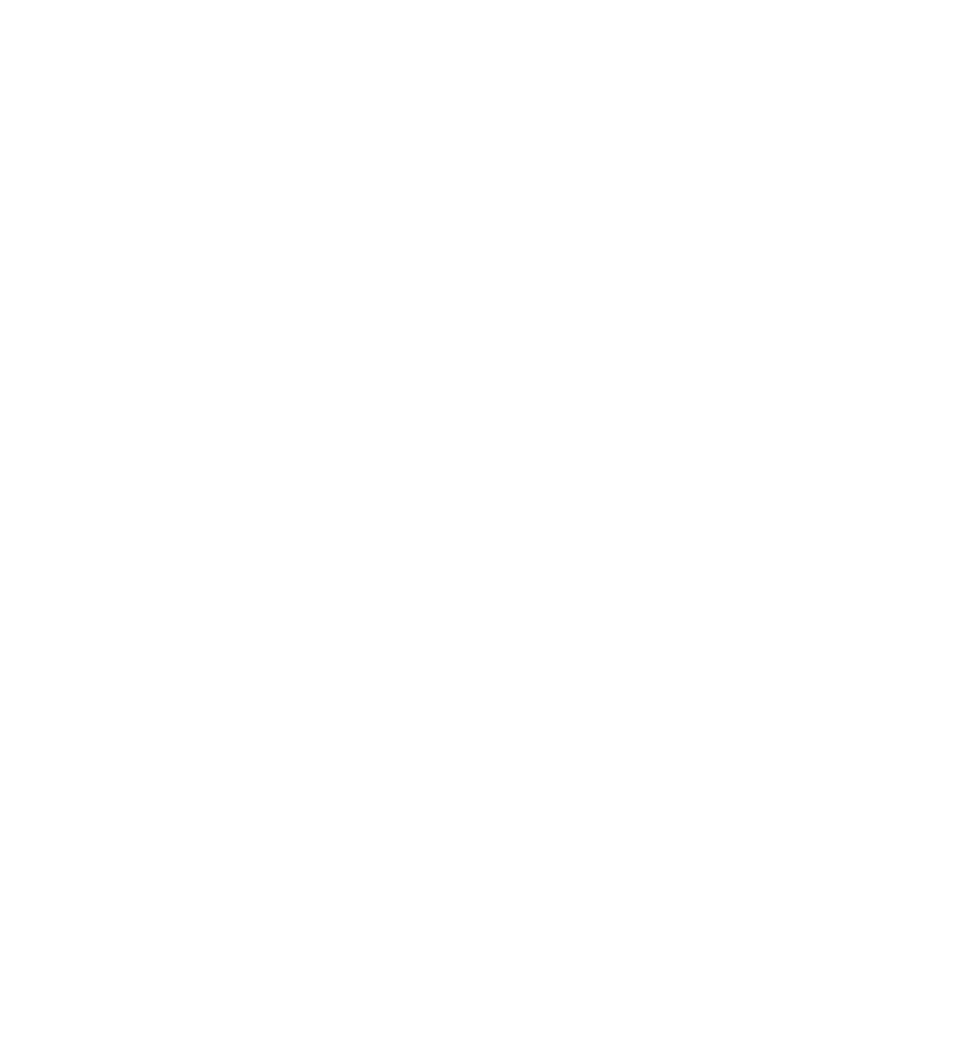 | 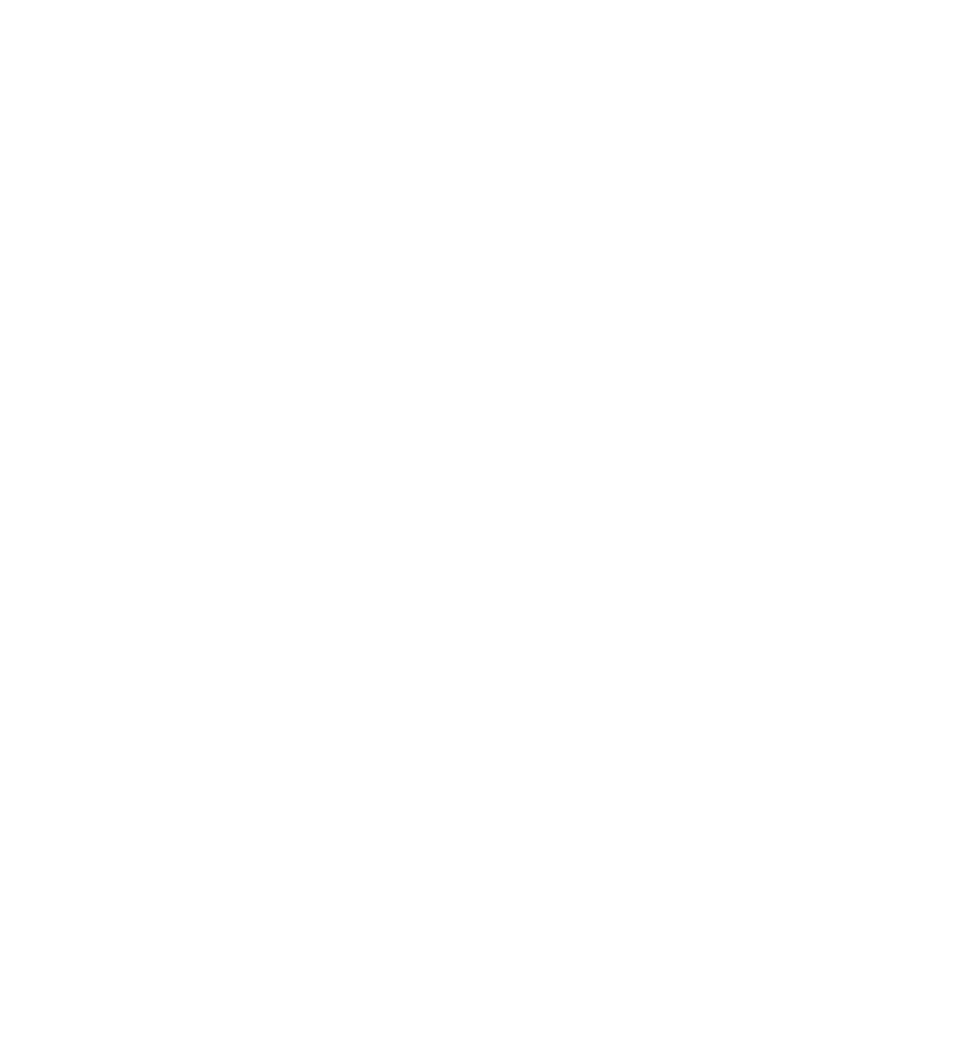 | Extremely satisfied |

**
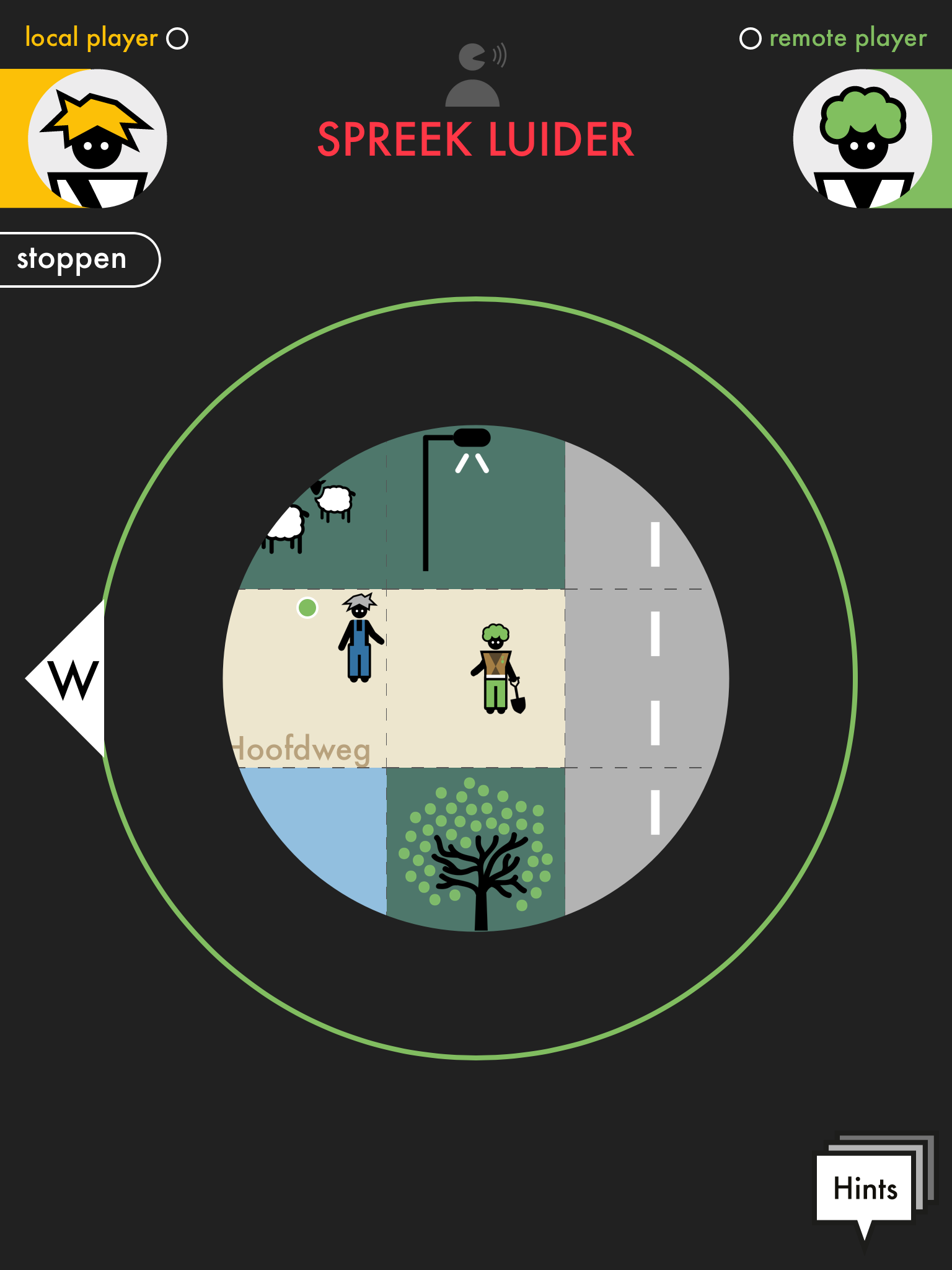
**

**Did you notice the feedback on the pitch and loudness often?**

With this we mean the resizing circle and the notifications ‘Speak louder’ and ‘Speak lower’ from the above screenshot.

|  |
| --- |

**How did you experience the precision of the feedback?**

|  |
| --- |

**What were you able to do with the feedback?**

|  |
| --- |


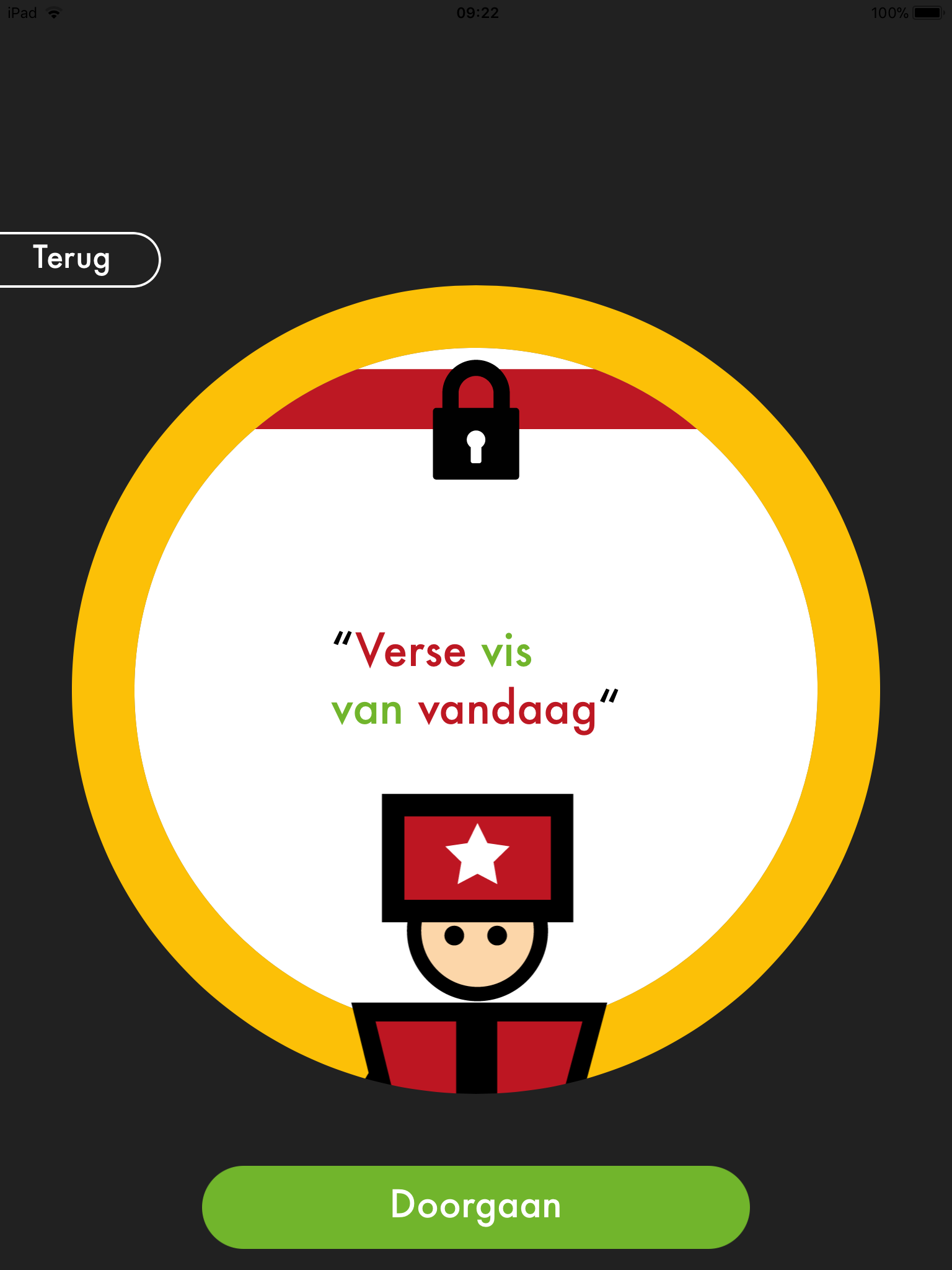


**How did you experience the feedback on pronunciation?**

With this we mean the red/green coloring in the pronounced key phrases.

|  |
| --- |

**To what extent did you disagree with this feedback?**

For example, words that were marked red but pronounced correctly according to you.

|  |
| --- |

**How did you generally experience playing Treasure Hunters?**

Think of positive and negative aspects.

|  |
| --- |
